# Proteomic Characterization of Canine Gastric Fluid by Liquid Chromatography–Mass Spectrometry for Development of Protein Biomarkers in Regurgitation, Vomiting, and Cough

**DOI:** 10.3389/fvets.2021.670007

**Published:** 2021-07-07

**Authors:** Megan Grobman, Hansjörg Rindt, Carol R. Reinero

**Affiliations:** ^1^Department of Clinical Sciences, College of Veterinary Medicine, Auburn University, Auburn, AL, United States; ^2^Department of Veterinary Medicine and Surgery, College of Veterinary Medicine, University of Missouri, Columbia, MO, United States

**Keywords:** reflux, aspiration, extra-esophageal, oropharyngeal, respiratory

## Abstract

Reflux and aspiration in people cause and exacerbate respiratory diseases in the absence of gastrointestinal signs. Protein biomarkers in humans detect extraesophageal reflux (EER) from oropharyngeal (OP) and bronchoalveloar lavage samples. Reflux likely contributes to respiratory disease in dogs. The objectives of this study were to analyze the canine gastric fluid (GF) proteome and compare this to the OP proteome in normal, vomiting/regurgitating, and coughing dogs to identify biomarkers for EER/aspiration. Twenty-three client-owned dogs were enrolled. Canine GF samples (*n* = 5) and OP swabs in normal (*n* = 6), vomiting/regurgitating (*n* = 7), and coughing (*n* = 5) dogs were within 2 weeks of sample collection. Protein digests were analyzed by liquid chromatography–mass spectrometry. Differential abundance (DA) of proteins between groups was evaluated by Fisher's exact test with *p* < 0.0004 significance level after correction for multiple comparisons. DA was found between all groups (*p* < 0.0001): GF vs. normal (*n* = 130 proteins), coughing vs. normal (*n* = 22 proteins), and vomiting/regurgitating vs. normal (*n* = 20 proteins). Protein abundance was highly variable between dogs. Gastrointestinal-specific proteins were found in OP swabs from vomiting/regurgitating and coughing dogs but not from healthy dogs. In conclusion, the proteomic composition of the OP varies between health and disease. The presence of gastrointestinal-specific proteins in OP of coughing dogs may suggest reflux and/or aspiration as contributing factors. The variable protein abundance warrants investigation into biomarker panels.

## Introduction

Biomarkers diagnose, monitor, and identify populations at risk for disease. Biomarkers also have the potential to advance the understanding of disease pathogenesis by allowing an objective investigation of the efficacy of novel therapeutics (1–5). In recent years, the development and implementation of biomarkers have been making an increasingly large impact in companion animals (2, 5).

Reflux of gastrointestinal contents is a source of acute and chronic pulmonary disease, with a prevalence of 50% in humans with chronic cough, asthma, chronic obstructive pulmonary disease, and idiopathic pulmonary fibrosis (6–10). Reflux and aspiration are implicated in canine respiratory disorders including rhinitis, laryngeal dysfunction, and upper/lower airway and pulmonary parenchymal diseases (11–13). Despite the implied association between reflux, aspiration, and respiratory disease, a non-invasive widely available means of detecting reflux and aspiration in dogs is needed.

The two major types of reflux implicated in the development of respiratory disease are gastroesophageal reflux (GER) and extraesophageal reflux (EER). Gastroesophageal reflux refers to reflux of gastric contents into the esophagus, while EER refers to refluxate reaching structures beyond the esophagus, including the oro- and nasopharynx, larynx, and airways (14). Reflux reaching the nasopharynx and larynx are referred to as nasopharyngeal reflux and laryngopharyngeal reflux, respectively. The severity of GER and the prevalence of EER are correlated (15). In people, EER increases the risk of macro- and microaspiration, laryngeal dysfunction, and exacerbation of pulmonary pathology (14, 15). Importantly, treatment of EER in people reduces the frequency of respiratory disease exacerbation and slows the rate of decline in lung function (7). This makes identifying patients with reflux-associated respiratory disease critically important.

Human gastric fluid (GF) has been extensively characterized, allowing for the identification of reflux biomarkers outside the gut (16–18). Gastric pepsin (pepsin A), a component of refluxate identified in humans with EER, is a biomarker in oral and respiratory secretions with improved sensitivity (100%) over ambulatory esophageal pH monitoring (63%) (16, 18–21). Though proteomic evaluation has been performed for several biological fluids in dogs (3, 22), characterization of the canine GF proteome has not been performed previously.

The objectives of this pilot study were to characterize the protein composition of canine GF using liquid chromatography–mass spectrometry (LC–MS) and compare the GF proteomic profile to that of the OP in healthy, vomiting/regurgitating, and coughing dogs to detect potential biomarkers of EER. We hypothesized that by using a high-coverage proteome analysis we could identify proteins relevant as potential biomarkers of canine extraesophageal reflux disease (EERD).

## Materials and Methods

### Animals

Twenty-three companion dogs presenting to the University of Missouri Veterinary Health Center were prospectively enrolled with informed owner consent (University of Missouri IACUC #9154). Residual canine GF was collected directly *via* nasogastric or orogastric tubes in patients for whom these interventions were deemed medically necessary by the attending clinician. Sampling of gastric fluid from healthy dogs could not be ethically justified due to risk to the patient. Gastric fluid samples were collected prior to feeding (≥6 h). Oropharyngeal swabs were collected from healthy dogs, dogs with a history of regurgitation/vomiting, and coughing dogs. The healthy dogs had an unremarkable physical examination and no clinical evidence of gastrointestinal (GI) or respiratory disease within the preceding 6 months. The regurgitating/vomiting dogs were enrolled if they had a documented episode of regurgitation or vomiting within 12 h of sample collection. The coughing dogs were enrolled if they had a history of cough for >2 weeks without a clinical evidence of GI disease (e.g., vomiting and regurgitation). Dogs were excluded from the study if they had a mixed clinical evidence of respiratory and GI disease or if they were receiving prokinetics, antacids, antibiotics, or probiotics at least 2 weeks before the sample collection. Dogs with a gag at the end of a cough paroxysm were not considered to have a GI disease and were therefore not excluded.

### Sample Collection

Two milliliters of GF was collected *via* nasogastric or orogastric tubes and placed in a sterile red top blood collection tube. Samples were collected from unsedated dogs using minimum physical restraint needed for sample collection. Oropharyngeal swabs, collected by vigorously rubbing this region in each gently restrained dog meeting our inclusion criteria, were placed in 2 ml of sterile saline in a red top tube as mentioned earlier. The samples were gently vortexed and stored in polypropylene conical bottom Eppendorf tubes (Fischer Scientific, Chicago, IL) at −20°C. All samples were evaluated within 2 weeks of collection.

### Proteome Analysis

Proteome analysis was performed by the Gehrke Proteomics Center at the University of Missouri. Protein was extracted using four volumes of ice-cold acetone. Following washing with 80% acetone (in water), the pellet was re-suspended with 6 M urea, 2 M thiourea, and 100 mM ammonium bicarbonate. Protein was quantified using the EZQ assay according to the manufacturer's instructions (Invitrogen/Life Technologies). After quantification, the protein amount was normalized across all samples by dilution in urea buffer. Twenty-five micrograms of protein from each sample was digested with trypsin. The resulting peptides were desalted using C18 pipette tips (Pierce/Thermo), lyophilized, and re-suspended in 25 μl of acetonitrile/formic acid (0.1%). Ten micrograms of the re-suspended peptides was loaded on a C8 trap column (Thermo PepMap 100, 5 cm × 300 um) and then separated using a 400-nl/min 70-min gradient on a self-packed C18 column (75 um × 20 cm × 1.7 um particles—Waters BEH C18) at 50°C in the CaptiveSpray nanospray source. The mobile phase consisted of 0.1% formic acid in water (A) and 0.1% formic acid in acetonitrile (B), and the LC gradient was for B: initially 2%, followed by 26-min ramp up to 17%, 17–25% over 36 min, 25–37% over 15 min, gradient of 37–80% over 6 min, and then held at 80% for 7 min, with a total run time of 90 min. MS + MS/MS data were acquired on a Proxeon Easy nLC system attached to an LTQ Orbitrap mass spectrometer using the parallel accumulation serial fragmentation (PASEF) (1) method over the 90-min gradient. Capillary voltage was set to 1,600 V, trapped ion mobility spectrometry (tims)-on, PASEF-on (10 PASEF frames, overlap of five; i.e., between MS acquisitions), and 100–1,700 m/z mass range. Cycle time was for 1 MS and 15 PASEF = 1.8 s (~120 MS/MS acquired per cycle). Repeated acquisition was threshold × 4 within 0.4 min. Active exclusion involved release after 1 min (exclude from MS/MS the same peptide mass, notwithstanding the criteria above, and release after 1 min of elution). MS data were collected over an m/z range of 100–1,700. During MS/MS data collection, each tims cycle included 1 MS + an average of 10 PASEF MS/MS scans.

### Data Analysis

The LTQ Orbitrap XL raw files were submitted to the Proteome Discoverer search engine for protein identification (V2.2.0.388; Sequest HT). The protein database for *Canis lupis familiaris* (50,035; last update 2/5/2019 sequences) was downloaded from NCBI using the search term “dogs.” Data were searched with the following parameters: trypsin (full) as enzyme, two missed cleavages allowed, six- to 150-amino-acid peptide length, 10 ppm mass tolerance on precursor ions, 0.6 Da on fragment ions, carbamidomethyl cysteine as a fixed modification, and oxidized methionine as variable. Spectrum grouper was used with the following parameters: precursor mass criterion, “same measure mass-to-charge”; precursor mass tolerance, 10 ppm; max RT difference (min), 1.5; allow mass analyzer mismatch, false; and allow MS order mismatch, false. The Percolator algorithm was used to automatically search a decoy database with the following criteria: target false discover rate (FDR, strict) 0.01, validation based on q-value. Proteins that passed this “high” confidence cutoff were examined further. Differential abundance (DA) of proteins was determined using PEAKS label-free quantitation based on the MS1 peak integration approach with the following parameters: algorithm: PEAKS-Q, retention time tolerance: 1 min, and mass tolerance: 40 ppm. Data were then filtered for 1% protein FDR with ≥1 unique peptide and ≥2-fold change. Spectral counting was used to determine differential protein abundance. Data were filtered for 1% protein FDR with ≥1 unique peptide and then >4 spectral counts per protein (mean of replicates). Protein abundance was described using the normalized spectral abundance factor (NSAF). Biological function was determined for the 200 most abundant proteins per group (GF, normal, vomiting/regurgitating, and coughing) as determined by NSAF. Biomarker candidates were evaluated according to their abundance in GF, tissue specificity, low or absent concentration in normal dog OP, and increased concentration in vomiting/regurgitating compared to normal dog OP. Candidate proteins were then assessed in coughing dogs to look for an evidence of EERD.

### Statistical Analysis

Statistical analysis was performed using Past (version 3.21) and SigmaPlot (version 14.0) data analysis software. Descriptive statistics were applied where appropriate as median (interquartile range, IQR). Differences in patient demographics between groups (GF, healthy, vomiting/regurgitating, and cough) were evaluated by Wilcoxon signed rank test with *p* ≤ 0.05 significance level. Principal component analysis was performed for all proteins identified across groups. Differential abundance of proteins between groups based on pairwise comparisons was evaluated by Fisher's exact test, with *p* < 0.0004 considered significant after correction for multiple comparisons. Coefficients of variation were calculated for each protein demonstrating a differential abundance between groups. A one-way analysis of similarity (ANOSIM) using the Bray–Curtis similarity index was used to test for similarity between groups based on the biological function of the detected proteins with *p* ≤ 0.001 significance level. Normality was evaluated by Shapiro–Wilk test. *Post-hoc* analysis (Dunn's method or Bonferroni correction for multiple comparisons) was applied where appropriate.

## Results

### Animals

Twenty-three companion dogs were prospectively enrolled. The breeds represented included mixed breed (*n* = 5), Labrador retriever (*n* = 3), Yorkshire terrier (*n* = 2), standard poodle (*n* = 2), and one each for Australian shepherd, Welsh corgi, basset hound, Jack Russel terrier, beagle dog, golden retriever, German Shepherd dog, Boston terrier, bull terrier, miniature poodle, and Catahoula leopard dog. Twelve dogs were spayed females, seven were castrated males, two were intact males, and two were intact females. No significant differences were detected between groups [GF (*n* = 5), healthy (*n* = 6), vomiting/regurgitating (*n* = 7), and coughing (*n* = 5)] for age, weight, or body condition score (BCS) (Table 1). These results were therefore grouped and are provided as range and median (IQR). The ages ranged from 6 months to 13 years, with a median (IQR) age of 9 years (7–10 years). The weights ranged from 1.7 to 43 kg, with a median (IQR) weight of 16.6 kg (9–29 kg). The body condition score (nine-point scale) ranged from 4 to 8, with a median (IQR) BCS of 5 (5–6) (23).

**Table 1:** Demographic data (age, weight, body condition score, sex and reproductive status) for each group are displayed as median (IQR).

	**Healthy** **(*n* = 6)**	**Coughing** **(*n* = 5)**	**Vomiting/regurgitating** **(*n* = 7)**	**Gastric fluid** **(*n* = 5)**
Age (years)	8.5 (7–12.3)	7 (3.5–10)	9 (8–10)	9 (9–10)
Weight (kg)	15.4 (11.2–16.5)	20 (4.5–26.2)	10.3 (9–25.5)	20.3 (18.9–33)
Body condition score (nine-point scale)	5.5 (5–6.25)	5 (4–5)	5 (4.5–6.5)	6 (5–6)
Intact females	0	0	2	0
Intact males	0	1	1	0
Spayed females	4	2	3	3
Castrated males	2	2	1	2

*Sex and reproductive status are also reported for each group. No statistically significant differences were detected between groups (p > 0.05)*.

### Proteomic Analysis

**Figure 1:**
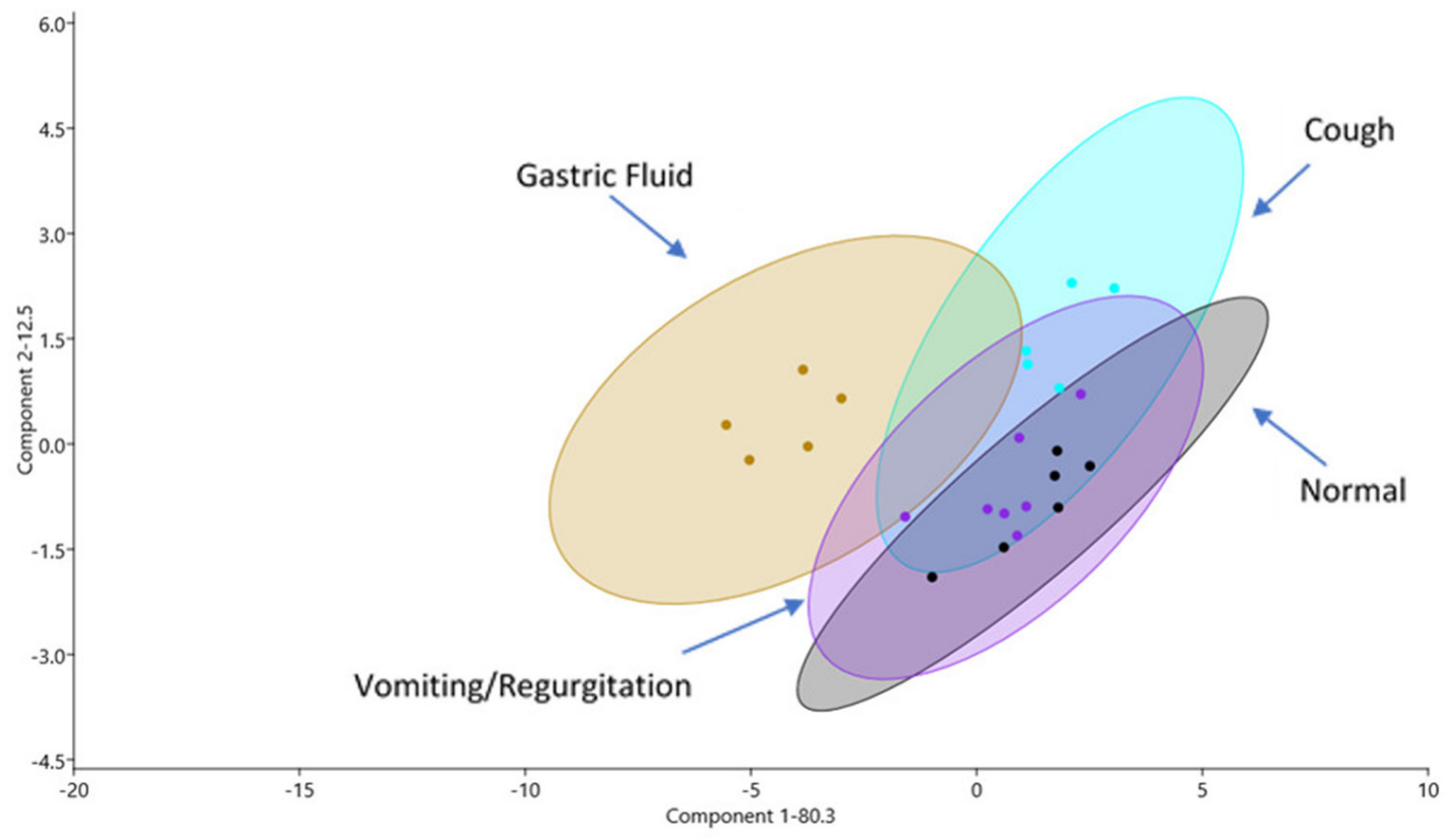
Proteomic profiles as shown *via* principal component (PC) analysis of samples for all four sample sites: GF (yellow), normal OP (gray), coughing OP (blue), and vomiting/regurgitating OP (purple). Similarities in proteomic profiles are demonstrated by overlapping regions on the PC1 vs. PC2 plot. Each dot represents an individual sample, and the ellipses represent 95% confidence intervals. GF, gastric fluid; OP, oropharyngeal.

From all samples, a total of 504 individual proteins were identified (Supplementary Materials). Within-group evaluation showed no significant differences for the total number of proteins identified. The median (IQR) number of proteins (per sample) per group were as follows: GF−122 proteins (110–230), healthy−259 proteins (203–309), vomiting/regurgitating−231 proteins (203–240), and cough−258 proteins (254–325). No significant differences between groups were found for the total number of proteins identified. The principal component analysis demonstrated an overlap between GF and OP swabs from vomiting/regurgitating and coughing dogs but not from normal dogs (Figure 1). Three pairwise comparisons were performed to identify DA proteins between groups: (1) normal vs. GF, (2) normal vs. vomiting/regurgitating, and (3) normal vs. cough.

#### Normal vs. GF

One hundred thirty proteins showed a significant DA (*p* <0.0004). Seventy proteins were significantly greater in abundance in GF; 60 were significantly greater in OP swabs from normal dogs. The coefficients of variation between dogs ranged from 16.9 to 244.9 for the DA proteins.

#### Normal vs. Vomiting/Regurgitating

Twenty proteins showed a significant DA (*p* < 0.0004). Thirteen proteins were significantly greater in abundance in OP swabs from vomiting/regurgitating dogs; seven were significantly greater in OP swabs from normal dogs. The coefficients of variation between dogs ranged from 45.5 to 264.6 for the DA proteins.

#### Normal vs. Cough

Twenty-two proteins showed a significant differential abundance (*p* < 0.0004). Twelve proteins were significantly greater in abundance in OP swabs from coughing dogs; 10 were significantly greater in OP swabs from normal dogs. The coefficients of variation between dogs ranged from 41.7 to 244.9 for DA proteins.

The OP proteome was also evaluated for GI-specific proteins. Gastrointestinal-specific proteins were defined as proteins that showed tissue specificity to the stomach, intestines, or pancreas. GI-specific proteins were found on OP swab in 0/6, 2/5, and 7/7 healthy, coughing, and regurgitating/vomiting dogs, respectively. Of the GI-specific proteins detected in OP, 4/6 were pancreatic in origin.

**Figure 2:**
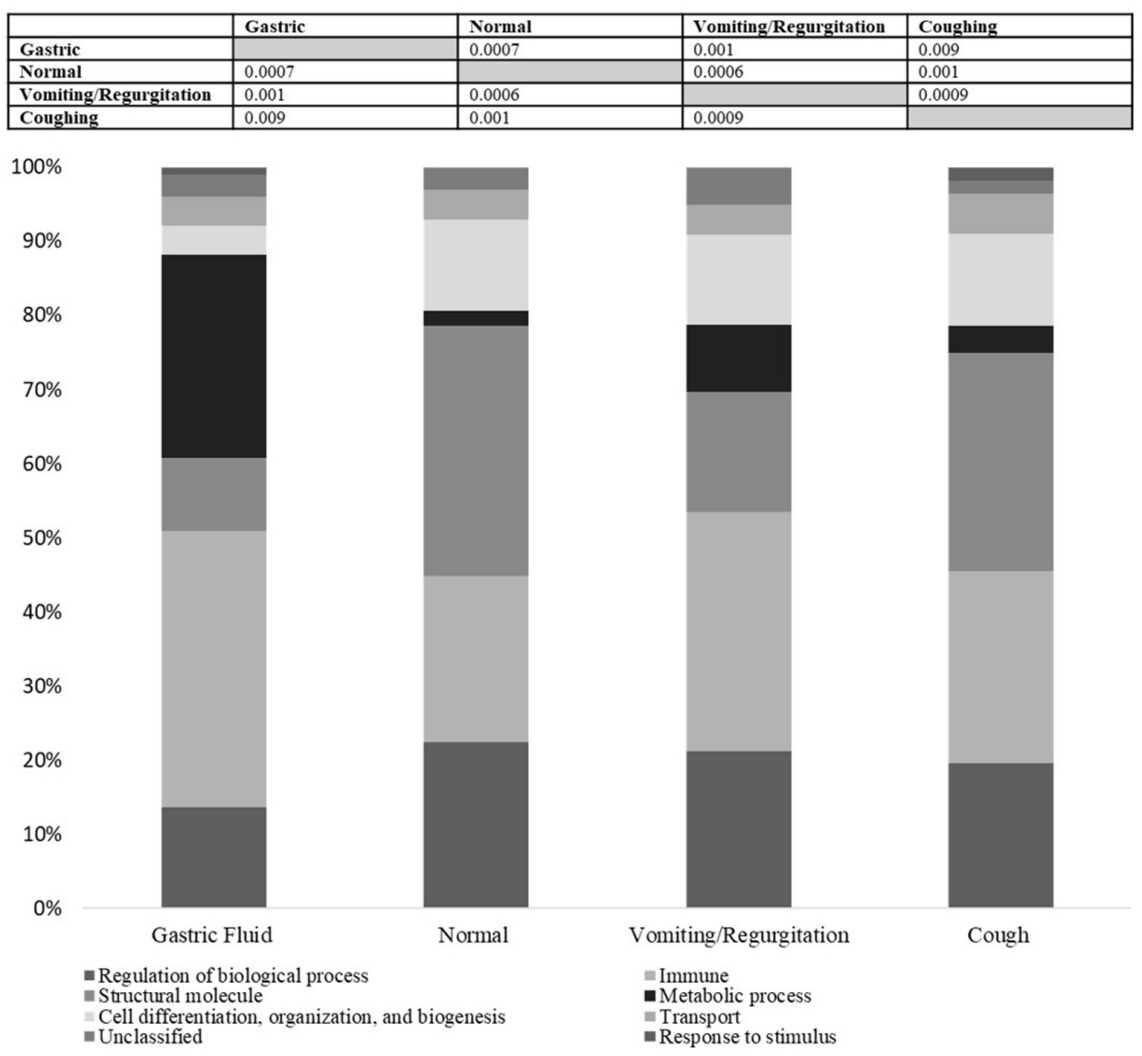
All groups were statistically dissimilar by ANOSIM based on the biological function of identified proteins (*p* ≤ 0.001). This suggests that the functional proteome differs by site (gastric fluid vs. oropharyngeal, OP) as well as between health and disease. The biological function profile for each group is displayed in the stacked bar chart above. Metabolic and immune proteins were over-represented in gastric fluid compared to normal dog OP swabs (*p* < 0.05). Metabolic proteins were over-represented in vomiting/regurgitating dog OP swabs compared to healthy dogs (*p* < 0.05) as were proteins with immunologic function (*p* < 0.05). Coughing dog OP swabs had increased the number of proteins involved in cellular differentiation and stimulus response compared to normal and regurgitating/vomiting dogs (*p* < 0.05).

**Figure 3:**
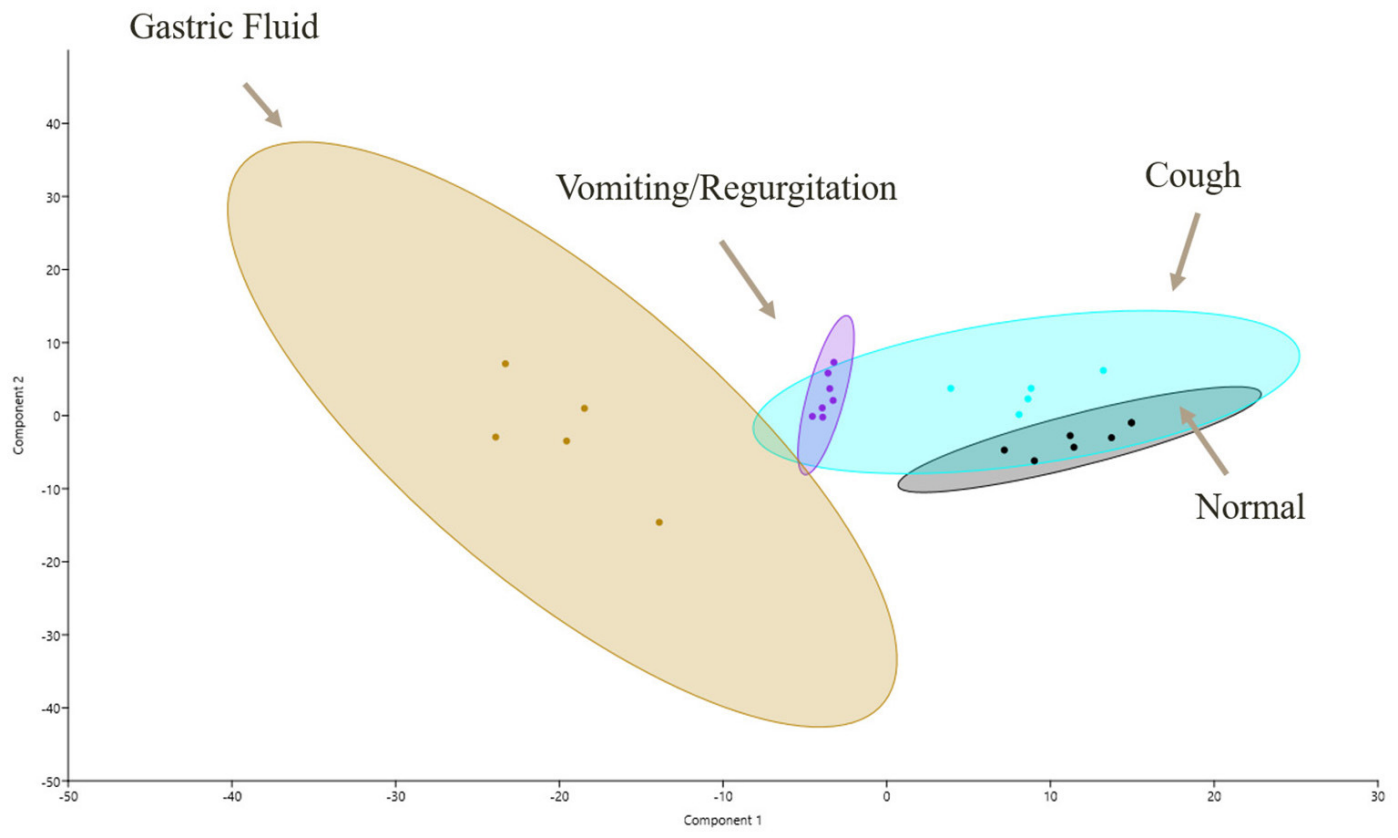
Functional proteomic profiles as shown *via* principal component (PC) analysis of samples for all four sample sites: GF (yellow), normal OP (gray), coughing OP (blue), and vomiting/regurgitating OP (purple). Similarities in proteomic profiles are demonstrated by overlapping regions on the PC1 vs. PC2 plot. Each dot represents an individual sample, and the ellipses represent 95% confidence intervals. GF, gastric fluid; OP, oropharyngeal.

For the biological function of proteins, all groups were found to be dissimilar on ANOSIM, with *p* ≤ 0.05 (Figure 2). The principal component analysis demonstrated an overlap between GF and OP swabs from vomiting/regurgitating and coughing dogs. No overlap was found between normal and vomiting/regurgitating dogs (Figure 3). The metabolic and immune proteins were over-represented in GF compared to normal dog OP swabs (*p* ≤ 0.05). Seventy-three percent of gastric metabolic proteins were pancreatic in origin, which is over-represented compared to salivary (14%), gastric (10%), and intestinal (3%) proteins (*p* ≤ 0.05). Pancreatic metabolic proteins were also increased in overall abundance compared to salivary, gastric, and intestinal metabolic proteins by NSAF. Pepsin A was identified in GF from all dogs but was less abundant compared to other non-gastric metabolic proteins (Table 2). Metabolic proteins were likewise over-represented in OP swabs in vomiting/regurgitating dogs compared to healthy dogs (*p* < 0.05) as were proteins with an immunologic function (*p* < 0.05). The coughing dogs had increased numbers of proteins involved in cellular differentiation and stimulus response compared to normal and regurgitating/vomiting dogs (*p* < 0.05).

**Table 2:** Metabolic proteins demonstrating increased abundance (GF vs. normal) and relative abundance in GF based on normalized spectral abundance factors are provided.

**Metabolic protein**	**Tissue of origin**	**Relative abundance (gastric fluid)**
Double-headed protease inhibitor	Salivary	1/230
Pancreatic lipase	Pancreatic	4/230
Chymotrypsin-C	Pancreatic	12/230
Pancreatic alpha-amylase	Pancreatic	16/230
Anionic trypsin	Pancreatic	19/230
Chymotrypsin	Pancreatic	37/230
Pancreatic secretory granule protein	Pancreatic	42/230
Chymotrypsin-like elastase	Pancreatic	46/230
Zymogen granule membrane-associated protein	Pancreatic	48/230
Zymogen membrane granule protein	Pancreatic	50/230
Pancreatic triacylglycerol lipase	Pancreatic	54/230
Gastric lipase	Gastric	58/230
Colipase	Pancreatic	63/230
Bile salt-activated lipase	Pancreatic	66/230
Chymosin	Intestinal	72/230
Pepsin A	Gastric	86/230

*Only metabolic proteins found within the 100 most abundant proteins are displayed*.

*GF, gastric fluid*.

## Discussion

In this pilot study, untargeted LC–MS was used to evaluate the canine GF proteome and identify several candidate biomarker proteins demonstrating DA in health and disease in the dog. Proteins specific to the GI tract were identified in the OP of vomiting/regurgitating and coughing dogs but not of healthy dogs. This suggests a potential role for reflux in the pathogenesis of respiratory disease in dogs and the possible utility of protein biomarkers as a screening tool for dogs with EERD and aspiration. Reflux and aspiration contribute significantly to the pathogenesis and progression of respiratory disease in people (6–9, 24). While a similar relationship is suspected in veterinary patients, a readily available diagnostic test capable of detecting EERD and aspiration is lacking (25), and the present proteomics approach shows promise. Though further studies are needed, significant between-dog variability may suggest an improved utility of biomarker panels over individual proteins.

Understanding the link between reflux and aspiration in clinical patients is critically important as people with a reflux-associated respiratory disease have demonstrated reductions in both disease exacerbation and declines in lung function following treatment targeting GERD and EERD (7). A similar targeted therapeutic approach may similarly be beneficial in dogs with airway, interstitial, and/or pulmonary parenchymal diseases. In experimental canine models, a link between respiratory disease, reflux, and aspiration in dogs has been demonstrated, with bronchoconstriction, laryngospasm, laryngeal paresis/paralysis, and microaspiration occurring in response to the application of acid and digestive enzymes to the esophagus and larynx (26–29). In companion dogs, this association is less clear. In brachycephalic dogs, an animal model for obstructive sleep apnea in people, treatment for presumptive GERD/EERD significantly improved the clinical signs of brachycephalic airway syndrome and minimized post-surgical complications (24, 25, 30). As such, treatment for EERD may also benefit dogs with other naturally developing respiratory disorders in which reflux and repetitive microaspiration play a role in disease development, progression, and exacerbation (12). Furthermore, similarities in anatomy, physiology, and several pathologic disorders make experimental and naturally occurring canine models integral to translational research in humans (31–33).

Identifying dogs with naturally occurring GERD and EERD poses a diagnostic challenge as reliable, inexpensive, and minimally invasive diagnostic tests are lacking. In people, biomarkers have been effectively utilized as a part of a multimodal approach to diagnosing reflux and aspiration-associated respiratory syndromes (AARS) (8, 18, 20, 34). Though biomarkers have been utilized effectively for a number of diseases in dogs, investigation into the biomarkers of reflux and aspiration has been rarely performed (2, 5, 13, 22). In this study, LC–MS was successfully used to generate an initial proteomic profile of canine GF to identify potential biomarker candidates. The principal component analysis demonstrated a complete separation, with a CI of 95%, of the GF proteome and normal dog OP proteome, suggesting that these two sites significantly differ with respect to their protein composition. This is a key initial finding supporting that, in the absence of reflux, there are no gastric proteins in the healthy dog OP and sets the stage to identify biomarker gastric proteins in this location in disease states.

Statistically significant DA for multiple proteins was identified between all groups compared to normal dogs. This finding suggests that the canine proteome differs not only by the site of collection [GF vs. OP (normal)] but also in health and disease (vomiting/regurgitating or coughing dogs vs. normal). The principal component analysis demonstrated an overlap between GF, vomiting/regurgitating, and coughing proteomes. Furthermore, GI-specific proteins were identified in dogs with vomiting/regurgitation as well as dogs with cough. No such proteins were found in healthy dogs. Though further studies investigating larger sample sizes are needed, this supports the hypothesis that GF biomarkers may be identified in vomiting/regurgitating and coughing dogs.

Gastric pepsin (pepsin A) is a component of the human gastric refluxate which has been used as a biomarker to identify patients with EER and aspiration (16, 18, 20, 21). While pepsin A was identified in canine GF, it was low in abundance. Furthermore, pepsin was not identified in OP swabs from any dog, including those with documented regurgitation/vomiting within 12 h of sample collection. It is possible that protein degradation, both *in vivo* and *ex vivo*, may have reduced the abundance of this protein below our limit of detection. The low abundance of pepsin A in GF and the lack of detection of pepsin A in OP swabs in vomiting/regurgitating dogs suggest this to be a less-than-ideal protein biomarker regardless of cause and represent a significant departure from the human literature (18, 34–38). Interestingly, pancreatic proteins were highly abundant in GF, likely suggesting that gastroduodenal reflux was a frequent occurrence in our population and supporting a biomarker study which identified bile in bronchoalveolar lavage fluid from dogs with pulmonary fibrosis (13). Salivary proteins were abundant in gastric fluid as well as in OP swabs in dogs with vomiting/regurgitation. While the amount of salivary proteins in gastric fluid may reflect the mode of collection (i.e., nasogastric or orogastric tube), it may also reflect the reflexive swallowing of salivary secretions (39). Increased abundance in dogs with vomiting/regurgitation may reflect bathing of the OP with concentrated salivary secretions from the gastric fluid, ptyalism secondary to nausea, or as a result of the esophagosalivary reflex which increases salivary secretions to protect against damage to the esophagus by gastric acid/digestive enzymes (40). A limitation of this study is the use of clinical dogs for the evaluation of gastric fluid. While evaluating healthy dogs would be ideal, the collection of gastric fluid could not be ethically justified in this population. Diet was not controlled in this population, other than all dogs received a commercial dog food. As such, the influence of diet on the GF proteome in dogs cannot be predicted. However, the fact that the documented differences in the proteome persist despite not controlling for diet may suggest that this is a true difference and not artifactual due to the influence of a common diet.

The presence of gastric proteins demonstrating an increased abundance in OP swabs from vomiting/regurgitating and coughing dogs compared to normal dogs opens doors for future biomarker validation studies in dogs with EERD and AARS. Important to future biomarker studies was the finding that the protein concentrations between dogs were extremely variable, echoing similar findings in people, thus suggesting limited utility for individual protein biomarkers and emphasizing the need for biomarker panels (41–43).

Biological protein function was determined for the 200 most abundant proteins per group, and a statistical evaluation by ANOSIM demonstrated that the OP swabs from normal, vomiting/regurgitating, and coughing dogs were statistically dissimilar, representing discrete functional proteomic profiles. This suggests that the differences in composition between groups may be enough to alter the functional resident proteome in health and disease. In vomiting/regurgitating dogs, proteins with metabolic (i.e., enzymatic) functions were found in increased abundance compared to those in normal dogs. This may reflect a contribution from the GF proteome, though the overall functional proteomic profiles were still considered dissimilar between these two groups.

## Data Availability Statement

The datasets presented in this study can be found in online repositories. The names of the repository/repositories and accession number(s) can be found below: massive.ucsd.edu (proteomxchange), identifier MSV000087178.

## Ethics Statement

The animal study was reviewed and approved by University of Missouri IACUC #9154. Written informed consent was obtained from the owners for the participation of their animals in this study.

## Author Contributions

MG, CR, and HR contributed to the writing of the manuscript. MG and HR were involved with sample preparation and data analysis. All authors contributed to the article and approved the submitted version.

## Conflict of Interest

The authors declare that the research was conducted in the absence of any commercial or financial relationships that could be construed as a potential conflict of interest.

## References

[1] Ranieri G Gadaleta CD Patruno R Zizzo N Daidone MG Hansson MG. A model of study for human cancer: spontaneous occurring tumors in dogs. Biological features and translation for new anticancer therapies. Crit Rev Oncol Hematol. (2013) 88:187–97. 10.1016/j.critrevonc.2013.03.005 23561333

[2] Ceciliani F Eckersall D Burchmore R Lecchi C. Proteomics in veterinary medicine: applications and trends in disease pathogenesis and diagnostics. Vet Pathol. (2014) 51:351–62. 10.1177/0300985813502819 24045891

[3] Miller I Presslmayer-Hartler A Wait R Hummel K Sensi C Eberini I. In between - proteomics of dog biological fluids. J Proteomics. (2014) 106:30–45. 10.1016/j.jprot.2014.04.016 24768907

[4] Aun MV Bonamichi-Santos R Arantes-Costa FM Kalil J Giavina-Bianchi P. Animal models of asthma: utility and limitations. J Asthma Allergy. (2017) 10:293. 10.2147/JAA.S121092 29158683 PMC5683778

[5] Myers MJ Smith ER Turfle PG. Biomarkers in Veterinary Medicine. Ann Rev Anim Biosci. (2017) 5:65–87. 10.1146/annurev-animal-021815-111431 27860493

[6] Irwin RS Zawacki JK Curley FJ French CL Hoffman PJ. Chronic cough as the sole presenting manifestation of gastroesophageal reflux. Am Rev Respir Dis. (1989) 140:1294–300. 10.1164/ajrccm/140.5.1294 2817591

[7] Celli BR Thomas NE Anderson JA Ferguson GT Jenkins CR Jones PW. Effect of pharmacotherapy on rate of decline of lung function in chronic obstructive pulmonary disease: results from the TORCH study. Am J Respir Crit Care Med. (2008) 178:332–8. 10.1164/rccm.200712-1869OC 18511702

[8] Gaude GS. Pulmonary manifestations of gastroesophageal reflux disease. Ann Thorac Med. (2009) 4:115–23. 10.4103/1817-1737.53347 19641641 PMC2714564

[9] Molyneux ID Morice AH. Airway reflux, cough and respiratory disease. Ther Adv Chronic Dis. (2011) 2:237–48. 10.1177/2040622311406464 23251752 PMC3513884

[10] Yuksel ES Vaezi MF. Extraesophageal manifestations of gastroesophageal reflux disease: cough, asthma, laryngitis, chest pain. Swiss Med Wkly. (2012) 142:w13544. 10.4414/smw.2012.13544 22442097

[11] Lux CN Archer TM Lunsford KV. Gastroesophageal reflux and laryngeal dysfunction in a dog. J Am Vet Med Assoc. (2012) 240:1100–103. 10.2460/javma.240.9.1100 22515631

[12] Nafe LA Grobman ME Masseau I Reinero CR. Aspiration-related respiratory syndromes in the dog. J Am Vet Med Assoc. (2016) 253:292–300. 10.2460/javma.253.3.292 30020014

[13] Määttä OM Laurila HP Holopainen S Lilja-Maula L Melamies M Viitanen SJ. Reflux aspiration in lungs of dogs with respiratory disease and in healthy west highland white terriers. J Vet Intern Med. (2018) 32:2074–81. 10.1111/jvim.15321 30311983 PMC6271311

[14] Koufman JA. Laryngopharyngeal reflux is different from classic gastroesophageal reflux disease. Ear Nose Throat J. (2002) 81:7–9. 12353431

[15] Groome M Cotton JP Borland M McLeod S Johnston DA Dillon JF. Prevalence of laryngopharyngeal reflux in a population with gastroesophageal reflux. Laryngoscope. (2007) 117:1424–8. 10.1097/MLG.0b013e31806865cf 17762271

[16] Krishnan U Mitchell JD Messina I Day AS Bohane TD. Assay of tracheal pepsin as a marker of reflux aspiration. J Pediatr Gastroenterol Nutr. (2002) 35:303–8. 10.1097/00005176-200209000-00012 12352517

[17] Kam SY Hennessy T Chua SC Gan CS Philp R Hon KK. Characterization of the human gastric fluid proteome reveals distinct pH-dependent protein profiles: implications for biomarker studies. J Proteome Res. (2011) 10:4535–46. 10.1021/pr200349z 21842849

[18] Hayat JO Gabieta-Somnez S Yazaki E Kang JY Woodcock A Dettmar P. Pepsin in saliva for the diagnosis of gastro-oesophageal reflux disease. Gut. (2015) 64:373–80. 10.1136/gutjnl-2014-307049 24812000

[19] Potluri S Friedenberg F Parkman HP Chang A MacNeal R Manus C. Comparison of a salivary/sputum pepsin assay with 24-hour esophageal pH monitoring for detection of gastric reflux into the proximal esophagus, oropharynx, and lung. Dig Dis Sci. (2003) 48:1813–7. 10.1023/A:1025467600662 14561007

[20] Kahrilas PJ Kia L. Pepsin: a silent biomarker for reflux aspiration or an active player in extra-esophageal mucosal injury? Chest. (2015) 148:300–1. 10.1378/chest.15-0506 26238826

[21] Ocak E Kubat G Yorulmaz I. Immunoserologic pepsin detection in the saliva as a non-invasive rapid diagnostic test for laryngopharyngeal reflux. Balkan Med J. (2015) 32:46–50. 10.5152/balkanmedj.2015.15824 25759771 PMC4342137

[22] Fernandes M Rosa N Esteves E Correia MJ Arrais J Ribeiro P. CanisOme–The protein signatures of *Canis lupus* familiaris diseases. J Proteomics. (2016) 136:193–201. 10.1016/j.jprot.2016.01.005 26776818

[23] Mawby D Bartges J Moyers T Cottrell T Davignon A Laflamme D. Comparison of body fat estimates by dual-energy x-ray absorptiometry and deuterium oxide dilution in client-owned dogs. Compend Contin Educ Pract Vet. (2001) 23:70. 10.5326/0400109

[24] Lecoindre P Richard S. Digestive disorders associated with the chronic obstructive respiratory syndrome of brachycephalic dogs: 30 cases (1999-2001). Revue Méd Vét. (2004) 155:141–6.

[25] Poncet CM Dupre GP Freiche VG Bouvy BM. Long-term results of upper respiratory syndrome surgery and gastrointestinal tract medical treatment in 51 brachycephalic dogs. J Small Anim Pract. (2006) 47:137–42. 10.1111/j.1748-5827.2006.00057.x 16512845

[26] Loughlin CJ Koufman JA Averill DB Cummins MM Kim YJ Little JP. Acid-induced laryngospasm in a canine model. Laryngoscope. (1996) 106:1506–9. 10.1097/00005537-199612000-00012 8948612

[27] Praud JP. Upper airway reflexes in response to gastric reflux. Paediatr Respir Rev. (2010) 11:208–12. 10.1016/j.prrv.2010.07.001 21109178

[28] Smith JA Houghton LA. The oesophagus and cough: laryngo-pharyngeal reflux, microaspiration and vagal reflexes. Cough. (2013) 9:12. 10.1186/1745-9974-9-12 23590893 PMC3640905

[29] Takeuchi K Nagahama K. Animal model of acid-reflux esophagitis: pathogenic roles of acid/pepsin, prostaglandins, amino acids. Biomed Res Int. (2014) 2014:532594. 10.1155/2014/532594 24672789 PMC3929485

[30] Poncet C Dupre G Freiche V Estrada M Poubanne Y Bouvy B. Prevalence of gastrointestinal tract lesions in 73 brachycephalic dogs with upper respiratory syndrome. J Small Anim Pract. (2005) 46:273–9. 10.1111/j.1748-5827.2005.tb00320.x 15971897

[31] Gharaee-Kermani M Ullenbruch M Phan SH. Animal models of pulmonary fibrosis. In: Varga J Brenner DA Phan SH editors. Fibrosis Research: Methods and Protocols. Totowa, NJ: Humana Press (2005). p. 251–259. 10.1385/1-59259-940-0:251 16118457

[32] Wright JL Cosio M Churg A. Animal models of chronic obstructive pulmonary disease. Am J Physiol Lung Cell Mol Physiol. (2008) 295:L1–15. 10.1152/ajplung.90200.2008 18456796 PMC2494776

[33] Williams K Roman J. Studying human respiratory disease in animals–role of induced and naturally occurring models. J Pathol. (2016) 238:220–32. 10.1002/path.4658 26467890

[34] Johnston N Dettmar PW Bishwokarma B Lively MO Koufman JA. Activity/stability of human pepsin: implications for reflux attributed laryngeal disease. Laryngoscope. (2007) 117:1036–9. 10.1097/MLG.0b013e31804154c3 17417109

[35] Lilja-Maula LI Palviainen MJ Heikkilä HP Raekallio MR Rajamäki MM. Proteomic analysis of bronchoalveolar lavage fluid samples obtained from west highland white terriers with idiopathic pulmonary fibrosis, dogs with chronic bronchitis, healthy dogs. Am J Vet Res. (2013) 74:148–54. 10.2460/ajvr.74.1.148 23270360

[36] Knight J Lively MO Johnston N Dettmar PW Koufman JA. Sensitive pepsin immunoassay for detection of laryngopharyngeal reflux. Laryngoscope. (2005) 115:1473–8. 10.1097/01.mlg.0000172043.51871.d9 16094128

[37] Grabowski M Kasran A Seys S Pauwels A Medrala W Dupont L. Pepsin and bile acids in induced sputum of chronic cough patients. Respir Med. (2011) 105:1257–61. 10.1016/j.rmed.2011.04.015 21592756

[38] Bardhan KD Strugala V Dettmar PW. Reflux revisited: advancing the role of pepsin. Int J Otolaryngol. (2012) 2012:646901. 10.1155/2012/646901 22242022 PMC3216344

[39] Lambert E. Secretion management. In: Ongkasuwan J Chiou E editors. Pediatric Dysphagia. Cham: Springer (2018). p. 255–69. 10.1007/978-3-319-97025-7_20

[40] Yandrapu H Marcinkiewicz M Sarosiek I Sarosiek J Poplawski C Han K. Role of saliva in esophageal defense: implications in patients with nonerosive reflux disease. Am J Med Sci. (2015) 349:385–91. 10.1097/MAJ.0000000000000443 25789686 PMC4418785

[41] Hu S Loo JA Wong DT. Human body fluid proteome analysis. Proteomics. (2006) 6:6326–53. 10.1002/pmic.200600284 17083142 PMC2910092

[42] Borrebaeck CA Wingren C. Transferring proteomic discoveries into clinical practice. Expert Rev Proteomics. (2009) 6:11–3. 10.1586/14789450.6.1.11 19210121

[43] Frantzi M Bhat A Latosinska A. Clinical proteomic biomarkers: relevant issues on study design and technical considerations in biomarker development. Clin Transl Med. (2014) 3:7. 10.1186/2001-1326-3-7 24679154 PMC3994249

