# Supplementary material for: Proteomic Characterization of Canine Gastric Fluid by Liquid Chromatography–Mass Spectrometry for Development of Protein Biomarkers in Regurgitation, Vomiting, and Cough
Source: Front Vet Sci. 2021 Jul 7;8:670007. doi: 10.3389/fvets.2021.670007 (PMC8292676; doi:10.3389/fvets.2021.670007)
Supplement: Supplementary file 1 [file Data_Sheet_1.PDF]

Identified Proteins	Molecular Weight	Healthy			Cough			Regurgitating/Vomiting			Gastric Fluid		
(Canis lupus familiaris)	(kDa)	Median (NSAF)	Q1	Q3	Median (NSAF)	Q1	Q3	Median (NSAF)	Q1	Q3	Median (NSAF)	Q1	Q3
Serum albumin	69	0.65	0.39	0.96	0.87	0.64	1.23	0.86	0.57	1.80	0.77	0.23	1.20
Keratin, type I cytoskeletal 13 isoform 1	49	1.06	0.95	1.14	1.22	0.96	1.35	0.94	0.85	0.98	0.29	0.18	0.37
IgGFc-binding protein	316	0.11	0.08	0.14	0.14	0.09	0.15	0.16	0.07	0.19	0.09	0.07	0.12
Mucin-5B	539	0.07	0.05	0.08	0.13	0.04	0.17	0.10	0.06	0.13	0.01	0.01	0.03
Keratin, type II cytoskeletal 6A isoform X4	61	0.78	0.76	0.86	0.98	0.64	1.02	0.79	0.52	0.89	0.02	0.00	0.08
Keratin, type II cytoskeletal 4	64	0.95	0.82	1.04	0.95	0.73	1.00	0.83	0.73	0.93	0.19	0.11	0.28
Lactotransferrin isoform 1	77	0.44	0.31	0.52	0.48	0.35	0.55	0.44	0.34	0.47	0.10	0.06	0.52
Keratin, type II cytoskeletal 5 isoform X2	62	0.76	0.67	0.84	0.89	0.61	0.94	0.69	0.53	0.85	0.13	0.05	0.29
Polymeric immunoglobulin receptor	83	0.32	0.20	0.48	0.20	0.19	0.36	0.24	0.18	0.37	0.18	0.16	0.22
Keratin, type II cuticular Hb4 isoform 1	63	0.40	0.29	0.55	0.67	0.48	0.71	0.43	0.18	0.62	0.00	0.00	0.05
Serotransferrin isoform 1	85	0.21	0.19	0.23	0.18	0.18	0.46	0.16	0.15	0.45	0.36	0.11	0.38
Actin, cytoplasmic 1	42	0.56	0.53	0.61	0.62	0.55	0.67	0.64	0.43	0.67	0.10	0.07	0.19
Lysozyme C	17	1.15	0.78	1.47	0.65	0.47	0.71	1.18	0.59	1.76	0.53	0.41	0.65
Ig:SUBUNIT=alpha	37	0.61	0.53	0.86	0.43	0.32	0.54	0.54	0.36	0.55	0.54	0.35	0.70
Angiopoietin-related protein 5-like	22	0.84	0.67	0.91	0.68	0.64	0.77	0.91	0.59	1.34	0.23	0.05	0.68
Mucin-19-like	86	0.10	0.06	0.15	0.24	0.20	0.30	0.23	0.08	0.38	0.05	0.05	0.08
Ovostatin homolog 2-like isoform X1	164	0.13	0.10	0.14	0.11	0.11	0.16	0.11	0.08	0.14	0.03	0.02	0.06
Deleted in malignant brain tumors 1 protein isoform X1	233	0.07	0.05	0.07	0.08	0.06	0.09	0.08	0.07	0.09	0.02	0.00	0.03
Keratin, type I cytoskeletal 16	52	0.68	0.49	0.95	0.83	0.52	0.96	0.65	0.42	0.78	0.08	0.00	0.13
Desmoplakin	332	0.05	0.03	0.08	0.08	0.02	0.08	0.02	0.02	0.07	0.00	0.00	0.00
Keratin 14	52	0.50	0.39	0.60	0.67	0.35	0.69	0.46	0.32	0.48	0.00	0.00	0.08
Annexin A1	39	0.35	0.33	0.51	0.36	0.33	0.38	0.38	0.37	0.62	0.05	0.03	0.10
Keratin, type I cuticular Ha6	52	0.29	0.17	0.30	0.40	0.38	0.40	0.35	0.13	0.41	0.00	0.00	0.00
Annexin 2	39	0.45	0.42	0.46	0.41	0.38	0.44	0.41	0.37	0.55	0.08	0.08	0.10

Lipocalin-Can f 6 allergen	22	0.64	0.64	0.67	0.91	0.77	0.91	0.45	0.34	0.55	0.14	0.09	0.32
Complement C3	180	0.02	0.01	0.04	0.06	0.05	0.16	0.06	0.01	0.08	0.09	0.01	0.15
BPI fold-containing family A member 2 isoform 1	27	0.13	0.08	0.15	0.59	0.19	0.78	0.11	0.09	0.28	0.81	0.67	0.85
Chain B, Crystal Structure Of Hemoglobin	16	0.19	0.19	0.28	0.50	0.44	0.69	0.19	0.16	0.41	0.38	0.19	1.13
Immunoglobulin lambda-like polypeptide 5-like isoform X2	25	0.42	0.26	0.52	0.48	0.40	0.60	0.40	0.30	0.48	0.48	0.48	0.56
Leukocyte elastase inhibitor	50	0.32	0.29	0.34	0.22	0.20	0.40	0.20	0.13	0.32	0.08	0.02	0.10
Keratin, type II cytoskeletal 2 oral isoform X1	66	0.55	0.47	0.66	0.71	0.47	0.73	0.48	0.37	0.70	0.08	0.00	0.21
BPI fold-containing family B member 1 isoform X2	54	0.19	0.17	0.24	0.35	0.24	0.39	0.17	0.14	0.23	0.19	0.11	0.22
Can f 2	20	0.60	0.44	0.69	0.75	0.65	1.15	0.45	0.40	0.53	0.20	0.10	0.30
Prolactin-inducible protein homolog	17	0.47	0.38	0.78	0.65	0.35	0.71	0.47	0.32	0.59	0.94	0.88	1.00
Can f 1	19	0.66	0.43	0.80	0.79	0.74	0.89	0.53	0.45	0.63	0.26	0.21	0.47
Lactoperoxidase isoform 2	80	0.13	0.08	0.17	0.10	0.09	0.18	0.14	0.06	0.20	0.03	0.01	0.23
Double-headed protease inhibitor, submandibular gland-like	16	0.19	0.14	0.23	0.25	0.19	0.69	0.56	0.09	1.00	0.56	0.13	1.06
Uncharacterized protein LOC488289	46	0.22	0.22	0.25	0.30	0.20	0.33	0.26	0.20	0.33	0.09	0.04	0.17
Myeloperoxidase	81	0.15	0.12	0.21	0.16	0.12	0.27	0.05	0.02	0.11	0.00	0.00	0.02
Epithelial keratin 1	64	0.37	0.31	0.47	0.42	0.39	0.50	0.30	0.27	0.41	0.09	0.09	0.11
Protein-glutamine gamma-glutamyltransferase E	78	0.17	0.13	0.20	0.10	0.08	0.13	0.12	0.06	0.15	0.00	0.00	0.00
Haptoglobin isoform 2	38	0.11	0.08	0.17	0.26	0.21	0.63	0.21	0.12	0.28	0.24	0.11	0.26
Unnamed protein product	52	0.11	0.06	0.16	0.13	0.06	0.17	0.12	0.09	0.18	0.15	0.02	0.19
Transketolase isoform X3	63	0.19	0.18	0.21	0.14	0.11	0.21	0.16	0.16	0.17	0.00	0.00	0.00
Keratin, type II cytoskeletal 78	56	0.21	0.17	0.23	0.18	0.09	0.23	0.21	0.18	0.23	0.00	0.00	0.00
Heat shock cognate 71 kDa protein	71	0.16	0.11	0.18	0.14	0.10	0.17	0.15	0.10	0.17	0.01	0.00	0.08
Alpha-enolase isoform 1	49	0.20	0.16	0.22	0.20	0.18	0.24	0.14	0.10	0.17	0.02	0.00	0.02
Kallikrein	29	0.33	0.31	0.37	0.34	0.28	0.34	0.28	0.22	0.29	0.21	0.17	0.34
Apolipoprotein A-I	30	0.22	0.09	0.27	0.43	0.37	0.50	0.47	0.10	0.50	0.10	0.00	0.27

Keratin, type I cytoskeletal 15 isoform X1	49	0.53	0.44	0.57	0.51	0.37	0.51	0.45	0.13	0.56	0.00	0.00	0.22
Keratin, type I cytoskeletal 24	56	0.11	0.07	0.18	0.30	0.07	0.34	0.13	0.05	0.20	0.00	0.00	0.00
Glyceraldehyde-3-phosphate dehydrogenase	36	0.19	0.19	0.19	0.19	0.17	0.22	0.25	0.15	0.26	0.06	0.06	0.08
Chain A, Recombinant Wild-Type Canine Milk Lysozyme (Apo-Type)	14	0.29	0.09	0.64	0.21	0.14	0.36	0.21	0.18	0.36	0.29	0.07	0.50
Alpha-actinin-4 isoform X12	107	0.04	0.00	0.07	0.08	0.07	0.09	0.04	0.01	0.08	0.01	0.00	0.02
Cysteine-rich secretory protein 2 isoform X1	30	0.15	0.03	0.39	0.13	0.07	0.20	0.23	0.08	0.27	0.13	0.10	0.17
Glucose-6-phosphate isomerase isoformX1	63	0.11	0.11	0.12	0.10	0.08	0.11	0.11	0.08	0.12	0.00	0.00	0.03
14-3-3 protein sigma	28	0.29	0.21	0.36	0.29	0.25	0.39	0.25	0.14	0.32	0.00	0.00	0.11
Cyclophilin A	17	0.38	0.31	0.46	0.35	0.35	0.47	0.53	0.47	0.62	0.06	0.00	0.18
Keratin, type II cuticular Hb6	53	0.05	0.01	0.06	0.08	0.00	0.21	0.09	0.04	0.25	0.00	0.00	0.00
Pancreatic alpha-amylase-like isoform X2	58	0.00	0.00	0.00	0.00	0.00	0.00	0.00	0.00	0.02	0.43	0.03	0.45
Fibrinogen beta chain isoform X3	56	0.04	0.02	0.08	0.09	0.07	0.09	0.05	0.04	0.19	0.05	0.02	0.14
Keratin, type I cytoskeletal 10	58	0.19	0.19	0.23	0.26	0.22	0.28	0.14	0.13	0.23	0.00	0.00	0.03
Carbonic anhydrase 6 isoform X1	37	0.07	0.03	0.11	0.14	0.08	0.27	0.08	0.03	0.12	0.16	0.00	0.22
Hyaluronidase-1 isoform X3	53	0.10	0.08	0.13	0.06	0.04	0.11	0.13	0.06	0.17	0.04	0.02	0.15
Immunoglobulin gamma heavy chain C	52	0.09	0.08	0.10	0.08	0.08	0.10	0.08	0.06	0.08	0.08	0.06	0.10
Mucin-7-like	28	0.11	0.04	0.17	0.32	0.11	0.32	0.18	0.11	0.27	0.18	0.11	0.21
Tubulin beta-4B chain-like	50	0.14	0.06	0.18	0.10	0.08	0.14	0.12	0.11	0.17	0.00	0.00	0.04
Fibrinogen gamma chain isoform X2	50	0.04	0.00	0.08	0.06	0.04	0.14	0.04	0.01	0.16	0.08	0.00	0.10
Zymogen granule protein 16 homolog B	18	0.33	0.25	0.33	0.22	0.22	0.33	0.28	0.25	0.50	0.33	0.22	0.39
Protein S100-A9 isoformX3	23	0.17	0.13	0.25	0.13	0.13	0.17	0.13	0.11	0.20	0.13	0.04	0.17
Pyruvate kinase PKM isoform X3	64	0.09	0.05	0.11	0.08	0.05	0.27	0.05	0.03	0.11	0.00	0.00	0.00
Serpin peptidase inhibitor, clade A (alpha-1 antiproteinase, antitrypsin), member 1 precursor	46	0.10	0.07	0.14	0.09	0.09	0.11	0.11	0.05	0.12	0.15	0.04	0.20
Peroxiredoxin-1	22	0.27	0.24	0.34	0.27	0.23	0.36	0.32	0.20	0.34	0.05	0.05	0.05

Triosephosphate isomerase	27	0.17	0.15	0.30	0.22	0.07	0.22	0.15	0.13	0.24	0.00	0.00	0.04
Immunoglobulin J chain	16	0.25	0.20	0.67	0.19	0.13	0.25	0.25	0.09	0.25	0.31	0.13	0.44
Keratin, type II cytoskeletal 3-like isoform 1	63	0.36	0.30	0.44	0.41	0.27	0.48	0.41	0.21	0.43	0.00	0.00	0.16
Periplakin isoform X1	205	0.01	0.00	0.03	0.01	0.01	0.03	0.04	0.01	0.05	0.00	0.00	0.00
Neuroblast differentiation-associated protein AHNAK	580	0.01	0.00	0.01	0.01	0.01	0.01	0.01	0.01	0.02	0.00	0.00	0.00
Chain A, Crystal Structure Of Hemoglobin From Dog (Canis Familiaris) At 3.5 Angstrom Resolution	15	0.10	0.07	0.13	0.27	0.20	0.53	0.27	0.03	0.33	0.20	0.00	0.33
Unnamed protein product	82	0.09	0.07	0.12	0.06	0.01	0.10	0.05	0.02	0.09	0.00	0.00	0.00
Arylsulfatase F	66	0.11	0.06	0.14	0.08	0.00	0.09	0.08	0.05	0.13	0.00	0.00	0.00
Submaxillary mucin-like protein-like	44	0.06	0.01	0.09	0.09	0.02	0.16	0.07	0.02	0.22	0.05	0.05	0.07
Serum albumin isoform X1	69	0.51	0.10	1.05	0.59	0.00	0.83	0.83	0.46	1.79	1.22	0.75	1.28
Plakophilin-1-like	81	0.06	0.03	0.07	0.09	0.01	0.12	0.02	0.02	0.09	0.00	0.00	0.00
Keratin, type II cytoskeletal 2 oral	54	0.15	0.11	0.23	0.24	0.09	0.28	0.19	0.13	0.23	0.00	0.00	0.00
Plastin-2 isoform X1	70	0.06	0.03	0.06	0.06	0.04	0.13	0.03	0.01	0.04	0.00	0.00	0.00
Histone H2B type 1-like isoform X2	14	0.07	0.02	0.23	0.29	0.07	0.79	0.29	0.18	0.39	0.00	0.00	0.07
Calcium-activated chloride channel regulator 1	100	0.00	0.00	0.00	0.00	0.00	0.00	0.00	0.00	0.01	0.06	0.04	0.14
Heat shock protein HSP 90-alpha, partial	73	0.05	0.03	0.05	0.07	0.05	0.08	0.05	0.04	0.08	0.01	0.00	0.01
Profilin-1	19	0.16	0.12	0.16	0.16	0.05	0.37	0.11	0.05	0.18	0.00	0.00	0.11
Transmembrane protease serine 9	58	0.03	0.03	0.06	0.07	0.05	0.14	0.02	0.01	0.04	0.00	0.00	0.02
Gelsolin	94	0.03	0.01	0.04	0.01	0.01	0.09	0.03	0.01	0.03	0.00	0.00	0.01
Heat shock protein beta-1	23	0.22	0.15	0.28	0.17	0.13	0.17	0.22	0.15	0.26	0.00	0.00	0.04
Chymotrypsin-C isoform 2	29	0.00	0.00	0.00	0.00	0.00	0.00	0.00	0.00	0.00	0.31	0.10	0.55
BPI fold-containing family B member 2	50	0.10	0.09	0.12	0.06	0.04	0.08	0.08	0.06	0.10	0.06	0.00	0.08
Tubulin alpha-1B chain	50	0.08	0.07	0.10	0.10	0.04	0.12	0.08	0.04	0.12	0.00	0.00	0.00
Ezrin isoform 1	69	0.05	0.04	0.07	0.07	0.04	0.09	0.04	0.04	0.05	0.01	0.00	0.04
Anionic trypsinogen	26	0.00	0.00	0.00	0.00	0.00	0.00	0.00	0.00	0.04	0.19	0.04	0.38

Actin, alpha skeletal muscle	42	0.02	0.01	0.02	0.02	0.00	0.50	0.02	0.01	0.24	0.00	0.00	0.00
ATP synthase subunit beta, mitochondrial isoform 1	56	0.07	0.04	0.08	0.07	0.07	0.09	0.05	0.05	0.10	0.05	0.02	0.05
14-3-3 protein zeta/delta isoform X3	28	0.18	0.18	0.21	0.18	0.14	0.29	0.18	0.14	0.18	0.00	0.00	0.07
Desmocollin type 2	91	0.05	0.03	0.07	0.03	0.02	0.05	0.02	0.01	0.05	0.00	0.00	0.00
S100 calcium binding protein A8	10	0.20	0.13	0.35	0.40	0.30	0.50	0.30	0.30	0.35	0.20	0.20	0.30
Epithelial keratin 2e	65	0.38	0.08	0.48	0.55	0.46	0.65	0.00	0.00	0.35	0.00	0.00	0.00
Keratin, type II cytoskeletal 6A	61	0.57	0.55	0.66	0.70	0.38	0.75	0.62	0.28	0.71	0.00	0.00	0.18
Pancreatic triacylglycerol lipase isoform 2	51	0.00	0.00	0.00	0.00	0.00	0.00	0.00	0.00	0.00	0.20	0.02	0.33
Desmoglein-1 precursor	114	0.05	0.04	0.07	0.04	0.02	0.04	0.01	0.00	0.02	0.00	0.00	0.00
Myosin-9	226	0.01	0.00	0.02	0.00	0.00	0.05	0.00	0.00	0.01	0.00	0.00	0.00
Thioredoxin domain-containing protein 5-like	25	0.12	0.06	0.12	0.16	0.12	0.20	0.12	0.10	0.16	0.04	0.04	0.04
Fibrinogen A-alpha-chain	97	0.00	0.00	0.01	0.02	0.02	0.02	0.01	0.00	0.04	0.02	0.00	0.02
L-lactate dehydrogenase A chain isoformX1	40	0.09	0.06	0.12	0.10	0.05	0.13	0.05	0.04	0.06	0.00	0.00	0.03
Inactive pancreatic lipase-related protein 1	51	0.00	0.00	0.00	0.00	0.00	0.00	0.00	0.00	0.00	0.08	0.00	0.45
Keratin, type I cuticular Ha1	45	0.02	0.01	0.11	0.00	0.00	0.00	0.02	0.00	0.08	0.00	0.00	0.00
Fast myosin heavy chain 2X	225	0.00	0.00	0.00	0.00	0.00	0.03	0.00	0.00	0.00	0.00	0.00	0.00
Macrophage-capping protein isoform X1	39	0.09	0.05	0.15	0.08	0.05	0.15	0.08	0.05	0.08	0.00	0.00	0.03
Transaldolase-like	38	0.13	0.09	0.13	0.11	0.03	0.13	0.08	0.05	0.12	0.00	0.00	0.00
Fructose-bisphosphate aldolase A isoform X3	45	0.03	0.00	0.07	0.11	0.04	0.18	0.07	0.04	0.09	0.00	0.00	0.00
Heat shock protein 70	70	0.04	0.00	0.08	0.06	0.03	0.09	0.04	0.01	0.06	0.00	0.00	0.00
Hemopexin	51	0.02	0.00	0.02	0.02	0.00	0.12	0.02	0.00	0.04	0.00	0.00	0.14
Bile salt-activated lipase isoform 1	76	0.00	0.00	0.00	0.00	0.00	0.00	0.00	0.00	0.00	0.11	0.00	0.18
Protein S100-A12	11	0.27	0.20	0.27	0.36	0.36	0.55	0.18	0.18	0.32	0.09	0.00	0.09
Secretoglobin family 1D member-like	10	0.05	0.00	0.10	0.30	0.10	0.40	0.10	0.00	0.10	0.40	0.30	0.40
Zinc-alpha-2-glycoprotein	36	0.08	0.04	0.13	0.06	0.03	0.08	0.14	0.04	0.15	0.00	0.00	0.08

Prostaglandin reductase 1 isoform X1	36	0.11	0.09	0.11	0.06	0.06	0.11	0.06	0.06	0.08	0.00	0.00	0.03
NAD(P)H dehydrogenase [quinone] 1	31	0.10	0.07	0.15	0.03	0.03	0.10	0.10	0.06	0.16	0.00	0.00	0.03
Cornulin	55	0.06	0.03	0.09	0.09	0.07	0.09	0.05	0.02	0.10	0.00	0.00	0.00
Cationic trypsin	26	0.00	0.00	0.00	0.00	0.00	0.00	0.00	0.00	0.04	0.35	0.15	0.46
Serpin B5 isoform X2	43	0.05	0.05	0.06	0.05	0.02	0.07	0.07	0.02	0.08	0.00	0.00	0.00
Glucose-6-phosphate 1-dehydrogenase isoform X1	64	0.06	0.04	0.08	0.05	0.05	0.08	0.03	0.01	0.05	0.00	0.00	0.02
78 kDa glucose-regulated protein isoform 5	72	0.08	0.05	0.08	0.06	0.06	0.06	0.06	0.03	0.07	0.00	0.00	0.00
Vitamin D-binding protein isoform X3	52	0.01	0.00	0.02	0.02	0.00	0.08	0.00	0.00	0.10	0.00	0.00	0.02
Pancreatic secretory granule membrane major glycoprotein GP2	57	0.00	0.00	0.03	0.00	0.00	0.02	0.00	0.00	0.01	0.09	0.04	0.18
Matrix metalloproteinase-9 isoform X1	78	0.02	0.00	0.03	0.03	0.01	0.15	0.00	0.00	0.01	0.00	0.00	0.00
Immunoglobulin heavy chain variable region, partial	15	0.07	0.02	0.12	0.07	0.07	0.07	0.07	0.03	0.17	0.07	0.00	0.07
Fibronectin, partial	268	0.00	0.00	0.00	0.00	0.00	0.00	0.00	0.00	0.00	0.01	0.00	0.04
Peroxiredoxin-6	25	0.16	0.13	0.19	0.08	0.04	0.08	0.04	0.02	0.10	0.00	0.00	0.04
Purine nucleoside phosphorylase isoform 1	32	0.09	0.04	0.15	0.09	0.09	0.16	0.06	0.06	0.11	0.00	0.00	0.00
Neutrophil collagenase	54	0.06	0.04	0.07	0.02	0.02	0.11	0.02	0.00	0.02	0.00	0.00	0.00
Unnamed protein product	231	0.01	0.01	0.02	0.00	0.00	0.01	0.01	0.01	0.02	0.00	0.00	0.00
Keratin, type II cytoskeletal 71	57	0.07	0.00	0.17	0.16	0.16	0.19	0.02	0.00	0.15	0.00	0.00	0.00
UPF0762 protein C6orf58 isoform X2	36	0.03	0.01	0.03	0.08	0.03	0.14	0.03	0.00	0.03	0.00	0.00	0.03
Cofilin-1-like	19	0.11	0.11	0.11	0.16	0.11	0.26	0.11	0.11	0.16	0.05	0.00	0.11
Protein disulfide-isomerase A3	57	0.03	0.02	0.05	0.05	0.04	0.05	0.04	0.00	0.06	0.02	0.00	0.04
Alpha-2-HS-glycoprotein	39	0.04	0.01	0.05	0.05	0.05	0.05	0.05	0.01	0.10	0.08	0.03	0.08
Annexin A8 isoformX1	37	0.09	0.04	0.13	0.08	0.08	0.11	0.03	0.01	0.07	0.00	0.00	0.00
Chymostrypsinogen B1-like precursor	28	0.00	0.00	0.00	0.00	0.00	0.00	0.00	0.00	0.00	0.21	0.04	0.36
Transgelin-2	22	0.09	0.09	0.13	0.09	0.09	0.14	0.05	0.05	0.14	0.05	0.00	0.14

Alpha-2-macroglobulin-like isoform X1	167	0.00	0.00	0.01	0.01	0.01	0.02	0.00	0.00	0.01	0.02	0.00	0.04
Protein disulfide-isomerase	57	0.04	0.01	0.05	0.05	0.02	0.05	0.04	0.01	0.05	0.00	0.00	0.04
Vimentin isoform 12	54	0.05	0.01	0.06	0.07	0.06	0.13	0.00	0.00	0.13	0.00	0.00	0.00
Malate dehydrogenase, mitochondrial-like	44	0.02	0.01	0.02	0.07	0.02	0.07	0.02	0.02	0.06	0.00	0.00	0.07
Retinol-binding protein 4	30	0.02	0.00	0.03	0.03	0.03	0.03	0.07	0.03	0.10	0.10	0.03	0.13
Neutrophil elastase	30	0.05	0.01	0.07	0.00	0.00	0.13	0.00	0.00	0.02	0.00	0.00	0.00
Serpin B10	45	0.06	0.04	0.07	0.07	0.04	0.07	0.04	0.02	0.06	0.00	0.00	0.02
Immunoglobulin heavy chain variable region, partial	16	0.06	0.02	0.06	0.06	0.06	0.06	0.06	0.06	0.06	0.06	0.00	0.06
Elongation factor 1-alpha 1	50	0.03	0.02	0.04	0.04	0.04	0.04	0.06	0.04	0.06	0.00	0.00	0.02
Xanthine dehydrogenase/oxidase isoform X2	147	0.01	0.01	0.02	0.01	0.01	0.01	0.01	0.00	0.01	0.00	0.00	0.00
Unnamed protein product	51	0.00	0.00	0.01	0.00	0.00	0.02	0.00	0.00	0.07	0.00	0.00	0.12
Uncharacterized protein LOC100855594	33	0.06	0.04	0.06	0.03	0.03	0.06	0.06	0.03	0.09	0.06	0.06	0.06
Malate dehydrogenase, cytoplasmic isoform 1	36	0.06	0.03	0.06	0.03	0.03	0.06	0.06	0.03	0.08	0.00	0.00	0.03
IQ motif containing GTPase activating protein 1	189	0.01	0.00	0.01	0.01	0.01	0.01	0.01	0.01	0.02	0.00	0.00	0.00
Zymogen granule membrane associated protein	48	0.00	0.00	0.00	0.00	0.00	0.00	0.00	0.00	0.00	0.13	0.00	0.21
Eukaryotic initiation factor 4A-I	46	0.04	0.03	0.06	0.04	0.04	0.04	0.04	0.02	0.07	0.02	0.02	0.02
Phosphoglycerate kinase 1 isoform 2	44	0.05	0.01	0.05	0.02	0.02	0.07	0.02	0.01	0.05	0.00	0.00	0.00
Carboxylesterase D1	62	0.01	0.00	0.03	0.03	0.02	0.03	0.02	0.01	0.04	0.00	0.00	0.00
ATP synthase subunit alpha, mitochondrial isoformX2	60	0.02	0.00	0.03	0.03	0.03	0.03	0.03	0.01	0.05	0.00	0.00	0.00
Glutathione S-transferase Mu 1 isoform 1	26	0.10	0.02	0.12	0.04	0.04	0.12	0.04	0.00	0.10	0.00	0.00	0.00
Carboxypeptidase A1 isoform X2	47	0.00	0.00	0.00	0.00	0.00	0.00	0.00	0.00	0.00	0.13	0.02	0.17
Keratin, type I cytoskeletal 17 isoform 1	48	0.32	0.06	0.49	0.27	0.21	0.50	0.44	0.00	0.49	0.00	0.00	0.00
Transthyretin isoform 2	16	0.00	0.00	0.05	0.19	0.13	0.19	0.06	0.00	0.22	0.06	0.00	0.06
Plasminogen isoform X1	91	0.00	0.00	0.01	0.00	0.00	0.02	0.00	0.00	0.01	0.02	0.01	0.09

Glycogen phosphorylase, muscle form isoform X2	93	0.02	0.01	0.04	0.01	0.00	0.04	0.00	0.00	0.02	0.00	0.00	0.01
Phosphatidylethanolamine-binding protein 1	21	0.12	0.10	0.14	0.10	0.10	0.14	0.05	0.05	0.07	0.00	0.00	0.00
Sulfide:quinone oxidoreductase, mitochondrial isoform X3	50	0.03	0.01	0.06	0.04	0.02	0.06	0.04	0.01	0.06	0.00	0.00	0.02
Ras-related protein Rab-10	23	0.04	0.01	0.08	0.04	0.04	0.04	0.00	0.00	0.04	0.00	0.00	0.00
Keratin-associated protein 13-2	19	0.03	0.00	0.05	0.00	0.00	0.11	0.11	0.00	0.24	0.00	0.00	0.00
Leucine-rich alpha-2-glycoprotein	39	0.04	0.00	0.15	0.00	0.00	0.03	0.03	0.00	0.03	0.00	0.00	0.00
Annexin A6 isoform X3	76	0.01	0.00	0.03	0.05	0.01	0.09	0.00	0.00	0.02	0.00	0.00	0.00
Rho GDP-dissociation inhibitor 2	23	0.04	0.04	0.08	0.04	0.00	0.22	0.04	0.00	0.09	0.00	0.00	0.00
Complement factor B	86	0.01	0.01	0.02	0.00	0.00	0.02	0.01	0.00	0.02	0.00	0.00	0.02
Trefoil factor family peptide 2	14	0.00	0.00	0.00	0.00	0.00	0.00	0.00	0.00	0.04	0.29	0.14	0.29
Mucin-4 isoform X2	131	0.02	0.01	0.03	0.01	0.01	0.02	0.01	0.00	0.01	0.00	0.00	0.00
Keratin, type II cytoskeletal 3-like	63	0.00	0.00	0.00	0.24	0.00	0.35	0.00	0.00	0.00	0.00	0.00	0.00
Elongation factor 2	95	0.01	0.00	0.02	0.03	0.01	0.03	0.02	0.01	0.03	0.00	0.00	0.00
Alpha-1-acid glycoprotein 1 isoform X1	23	0.04	0.04	0.08	0.04	0.04	0.09	0.04	0.04	0.04	0.00	0.00	0.00
Involucrin isoform X1	33	0.03	0.03	0.03	0.06	0.06	0.06	0.06	0.02	0.06	0.00	0.00	0.00
Guanine deaminase isoform X2	51	0.03	0.00	0.05	0.04	0.02	0.06	0.02	0.01	0.08	0.00	0.00	0.00
Catalase	60	0.00	0.00	0.03	0.02	0.00	0.08	0.00	0.00	0.00	0.00	0.00	0.00
Rab GDP dissociation inhibitor alpha	51	0.02	0.02	0.02	0.02	0.02	0.04	0.02	0.01	0.02	0.00	0.00	0.00
Adenylyl cyclase-associated protein 1 isoform X3	52	0.03	0.02	0.04	0.02	0.02	0.04	0.00	0.00	0.04	0.00	0.00	0.02
CD177 antigen	45	0.02	0.01	0.02	0.02	0.00	0.07	0.02	0.00	0.03	0.00	0.00	0.02
F-box only protein 50	30	0.10	0.08	0.10	0.07	0.00	0.07	0.03	0.00	0.08	0.00	0.00	0.00
SH3 domain binding glutamic acid-rich protein like 3	10	0.20	0.20	0.28	0.20	0.20	0.30	0.20	0.10	0.20	0.00	0.00	0.00
6-phosphogluconate dehydrogenase, decarboxylating isoform 1	53	0.02	0.02	0.05	0.04	0.02	0.06	0.02	0.00	0.02	0.00	0.00	0.00
Keratin, type II cytoskeletal 8	55	0.06	0.00	0.15	0.13	0.00	0.16	0.02	0.00	0.09	0.07	0.02	0.11
Complement C4-A	193	0.00	0.00	0.00	0.00	0.00	0.02	0.00	0.00	0.00	0.00	0.00	0.01

Chain A, Crystal Structure Of Dog Gastric Lipase In Complex With A Phosphonate Inhibitor	43	0.00	0.00	0.00	0.00	0.00	0.00	0.00	0.00	0.00	0.09	0.00	0.14
Chloride intracellular channel protein 1	27	0.04	0.04	0.06	0.07	0.04	0.07	0.04	0.00	0.04	0.00	0.00	0.07
Glutathione S-transferase omega-1 isoform 1	27	0.07	0.05	0.07	0.07	0.07	0.11	0.04	0.04	0.09	0.00	0.00	0.00
Immunoglobulin heavy chain constant region CH2	12	0.13	0.00	0.31	0.00	0.00	0.17	0.00	0.00	0.08	0.17	0.17	0.25
Mucin-5AC	304	0.00	0.00	0.00	0.00	0.00	0.00	0.00	0.00	0.00	0.01	0.01	0.04
Antithrombin-III isoform 1	52	0.00	0.00	0.00	0.00	0.00	0.00	0.00	0.00	0.05	0.04	0.02	0.06
Phosphoglycerate mutase 1 isoform 4	29	0.03	0.03	0.06	0.07	0.03	0.10	0.03	0.00	0.07	0.00	0.00	0.00
Calpain-1 catalytic subunit isoform 1	81	0.02	0.01	0.02	0.01	0.01	0.05	0.01	0.00	0.03	0.00	0.00	0.00
14-3-3 protein beta/alpha isoform X3	28	0.13	0.03	0.14	0.11	0.11	0.14	0.11	0.00	0.14	0.00	0.00	0.00
Immunoglobulin alpha heavy chain constant regin variant C	9	0.33	0.00	0.83	0.00	0.00	0.00	0.00	0.00	0.33	0.00	0.00	0.33
Keratin, type I cytoskeletal 25	49	0.01	0.00	0.02	0.08	0.02	0.10	0.00	0.00	0.04	0.00	0.00	0.00
Kininogen-1 isoform X3	44	0.00	0.00	0.00	0.02	0.02	0.02	0.00	0.00	0.03	0.00	0.00	0.09
Unnamed protein product	41	0.09	0.00	0.17	0.15	0.05	0.22	0.15	0.00	0.23	0.24	0.24	0.24
Family with sequence similarity 129, member B isoform X2	84	0.01	0.00	0.01	0.01	0.00	0.01	0.01	0.01	0.02	0.00	0.00	0.01
Probable methyltransferase BTM2 homolog isoform X3	13	0.00	0.00	0.06	0.08	0.00	0.15	0.08	0.04	0.15	0.00	0.00	0.00
Tropomyosin alpha-4 chain isoform X25	29	0.00	0.00	0.03	0.03	0.03	0.10	0.00	0.00	0.00	0.03	0.00	0.07
Calreticulin isoform 4	48	0.02	0.02	0.02	0.04	0.00	0.04	0.00	0.00	0.03	0.00	0.00	0.00
Filamin-A isoform 2	281	0.00	0.00	0.00	0.00	0.00	0.01	0.00	0.00	0.01	0.00	0.00	0.00
Carboxypeptidase A2 isoform 1	47	0.00	0.00	0.00	0.00	0.00	0.00	0.00	0.00	0.00	0.06	0.02	0.11
Olfactomedin-4	77	0.01	0.00	0.01	0.01	0.01	0.01	0.01	0.01	0.02	0.00	0.00	0.00
Annexin A3 isoform X3	54	0.01	0.00	0.02	0.02	0.02	0.07	0.04	0.01	0.04	0.00	0.00	0.02
Keratin, type I cytoskeletal 15	40	0.63	0.52	0.68	0.60	0.60	0.68	0.65	0.53	0.68	0.00	0.00	0.00
Sorcin isoform X1	35	0.01	0.00	0.03	0.06	0.03	0.09	0.03	0.00	0.04	0.00	0.00	0.00
Actin-related protein 3B isoform X2	42	0.01	0.00	0.04	0.02	0.00	0.12	0.00	0.00	0.04	0.00	0.00	0.00

Protein S100-A6 isoform 1	10	0.00	0.00	0.15	0.20	0.00	0.20	0.20	0.05	0.20	0.00	0.00	0.00
Coronin-1A isoform 1	51	0.02	0.00	0.03	0.00	0.00	0.08	0.00	0.00	0.02	0.00	0.00	0.00
Clusterin	52	0.03	0.00	0.04	0.02	0.00	0.02	0.02	0.01	0.02	0.00	0.00	0.02
Annexin A5	36	0.03	0.01	0.07	0.03	0.03	0.08	0.00	0.00	0.03	0.00	0.00	0.00
Ras-related protein Rab-7a	24	0.04	0.01	0.10	0.04	0.00	0.13	0.00	0.00	0.04	0.00	0.00	0.00
Prostaglandin-H2 D-isomerase isoform X1	33	0.00	0.00	0.02	0.00	0.00	0.03	0.03	0.00	0.03	0.00	0.00	0.03
Protein S100-A2 isoform X1	11	0.09	0.00	0.18	0.18	0.09	0.18	0.18	0.05	0.18	0.00	0.00	0.00
Immunoglobulin heavy chain variable region, partial	16	0.19	0.09	0.23	0.19	0.00	0.19	0.19	0.09	0.22	0.06	0.00	0.19
Hydroxyacyl-coenzyme A dehydrogenase, mitochondrial isoform X2	36	0.04	0.03	0.06	0.06	0.03	0.06	0.03	0.00	0.08	0.00	0.00	0.00
Phospholipase B domain containing 1	64	0.03	0.02	0.03	0.02	0.02	0.03	0.00	0.00	0.01	0.00	0.00	0.00
Protein FAM3D isoform X1	25	0.06	0.04	0.11	0.04	0.00	0.04	0.00	0.00	0.04	0.00	0.00	0.00
Immunoglobulin heavy chain variable region, partial	15	0.03	0.00	0.07	0.07	0.00	0.13	0.00	0.00	0.07	0.00	0.00	0.00
Immunoglobulin heavy chain constant region CH4	14	0.14	0.04	0.20	0.00	0.00	0.00	0.00	0.00	0.11	0.00	0.00	0.07
Protein-arginine deiminase type-4	74	0.01	0.00	0.01	0.03	0.01	0.07	0.00	0.00	0.00	0.00	0.00	0.00
Immunoglobulin kappa light chain variable region	14	0.11	0.07	0.14	0.07	0.07	0.07	0.07	0.07	0.07	0.07	0.07	0.07
Immunoglobulin lambda-like polypeptide 5-like isoform X1	25	0.44	0.35	0.53	0.48	0.24	0.52	0.28	0.12	0.42	0.48	0.00	0.56
Protein S100-A14 isoform X1	12	0.08	0.08	0.15	0.08	0.08	0.08	0.17	0.08	0.17	0.00	0.00	0.00
ADP/ATP translocase 2 isoform 2	33	0.00	0.00	0.02	0.03	0.00	0.03	0.03	0.02	0.03	0.00	0.00	0.03
Apolipoprotein H; beta-2-glycoprotein I	38	0.00	0.00	0.00	0.08	0.00	0.08	0.00	0.00	0.03	0.03	0.00	0.03
Myosin regulatory light polypeptide 9-like isoform X2	20	0.00	0.00	0.08	0.05	0.00	0.20	0.00	0.00	0.03	0.00	0.00	0.00
Serpin B6 isoform 2	44	0.01	0.00	0.04	0.02	0.00	0.02	0.02	0.00	0.02	0.00	0.00	0.05
Prelamin-A/C	74	0.01	0.00	0.01	0.01	0.01	0.01	0.01	0.01	0.02	0.00	0.00	0.00
Cornifin-A-like	10	0.10	0.03	0.10	0.20	0.10	0.40	0.00	0.00	0.10	0.00	0.00	0.00
Alpha-1B-glycoprotein	61	0.02	0.00	0.02	0.00	0.00	0.02	0.00	0.00	0.00	0.00	0.00	0.02

Creatine kinase U-type, mitochondrial isoform 1	47	0.02	0.01	0.02	0.02	0.02	0.02	0.02	0.01	0.02	0.02	0.00	0.04
Cathepsin D	44	0.02	0.01	0.02	0.02	0.02	0.02	0.02	0.01	0.03	0.02	0.00	0.02
Heat shock-related 70 kDa protein 2 isoform 1	70	0.08	0.06	0.09	0.06	0.00	0.07	0.09	0.04	0.10	0.00	0.00	0.00
Cathelicidin	19	0.05	0.05	0.05	0.05	0.00	0.21	0.00	0.00	0.00	0.00	0.00	0.00
Sciellin isoform X2	77	0.01	0.00	0.03	0.00	0.00	0.01	0.01	0.00	0.02	0.00	0.00	0.00
Histone H1.5-like	22	0.02	0.00	0.05	0.05	0.05	0.14	0.00	0.00	0.11	0.00	0.00	0.00
Keratin-associated protein 13-2	18	0.00	0.00	0.04	0.00	0.00	0.17	0.00	0.00	0.14	0.00	0.00	0.00
Phospholipase A2	16	0.00	0.00	0.00	0.00	0.00	0.00	0.00	0.00	0.00	0.19	0.06	0.44
Plastin-3 isoform X2	71	0.00	0.00	0.02	0.04	0.00	0.06	0.03	0.01	0.04	0.00	0.00	0.00
Actin-related protein 2 isoform 1	45	0.01	0.00	0.02	0.02	0.00	0.04	0.02	0.00	0.03	0.00	0.00	0.00
Allergen Can f 4 precursor	19	0.00	0.00	0.04	0.00	0.00	0.00	0.00	0.00	0.00	0.00	0.00	0.00
Alpha-2-macroglobulin isoform X2	165	0.00	0.00	0.00	0.00	0.00	0.02	0.00	0.00	0.00	0.00	0.00	0.00
Chymotrypsin-like elastase family, member 3B isoform 1	29	0.00	0.00	0.00	0.00	0.00	0.00	0.00	0.00	0.00	0.07	0.03	0.14
Actin-related protein 2/3 complex subunit 3	21	0.05	0.05	0.05	0.05	0.05	0.05	0.05	0.05	0.05	0.00	0.00	0.00
Protein S100-A10 isoform 1	11	0.09	0.09	0.09	0.09	0.00	0.18	0.09	0.00	0.18	0.00	0.00	0.00
Aldo-keto reductase family 1, member A1 (aldehyde reductase)	37	0.01	0.00	0.03	0.00	0.00	0.03	0.00	0.00	0.04	0.00	0.00	0.00
Ceruloplasmin isoform X2	126	0.00	0.00	0.00	0.00	0.00	0.01	0.00	0.00	0.00	0.00	0.00	0.02
Colipase (precursor)	12	0.00	0.00	0.00	0.00	0.00	0.00	0.00	0.00	0.00	0.42	0.08	0.50
Chain C, Crystal Structure Of Exportin-5:rangtp:pre-Mirna Complex	24	0.04	0.01	0.07	0.04	0.00	0.04	0.04	0.04	0.04	0.00	0.00	0.00
Heat shock protein HSP 90-beta isoformX1	82	0.01	0.01	0.01	0.02	0.02	0.04	0.04	0.01	0.04	0.00	0.00	0.00
Transitional endoplasmic reticulum ATPase isoform 3	89	0.01	0.00	0.01	0.01	0.01	0.02	0.00	0.00	0.02	0.00	0.00	0.00
Extracellular matrix protein 1	61	0.00	0.00	0.01	0.00	0.00	0.00	0.02	0.00	0.02	0.00	0.00	0.03
Myosin light polypeptide 6 isoform 1	17	0.00	0.00	0.00	0.06	0.06	0.12	0.00	0.00	0.03	0.00	0.00	0.00
Immunoglobulin heavy chain variable region, partial	15	0.00	0.00	0.00	0.00	0.00	0.00	0.00	0.00	0.00	0.00	0.00	0.07
Uteroglobin	10	0.00	0.00	0.00	0.00	0.00	0.10	0.00	0.00	0.05	0.00	0.00	0.10

CUB and zona pellucida-like domains 1 isoform 1	68	0.00	0.00	0.00	0.00	0.00	0.00	0.00	0.00	0.00	0.03	0.01	0.04
Chymotrypsinogen 2 isoform X2	28	0.00	0.00	0.00	0.00	0.00	0.00	0.00	0.00	0.00	0.18	0.00	0.21
Zymogen granule membrane protein 16 isoform X2	18	0.00	0.00	0.00	0.00	0.00	0.00	0.00	0.00	0.00	0.11	0.00	0.22
Voltage-dependent anion-selective channel protein 1	31	0.02	0.00	0.03	0.03	0.03	0.06	0.03	0.00	0.03	0.00	0.00	0.00
Keratin, type I cytoskeletal 42	54	0.07	0.00	0.16	0.19	0.00	0.30	0.22	0.01	0.27	0.00	0.00	0.00
Ubiquitin-60S ribosomal protein L40	15	0.03	0.00	0.07	0.07	0.07	0.07	0.00	0.00	0.07	0.00	0.00	0.00
Aldehyde dehydrogenase, mitochondrial isoform 2	57	0.00	0.00	0.01	0.02	0.00	0.02	0.00	0.00	0.03	0.00	0.00	0.00
Acyl-CoA synthetase short-chain family member 1	74	0.01	0.00	0.01	0.00	0.00	0.01	0.00	0.00	0.01	0.00	0.00	0.00
Keratin, type I cytoskeletal 19	44	0.00	0.00	0.00	0.00	0.00	0.02	0.00	0.00	0.16	0.00	0.00	0.16
Epithelial chloride channel protein-like	100	0.00	0.00	0.01	0.00	0.00	0.00	0.00	0.00	0.00	0.00	0.00	0.00
Keratin, type II cuticular Hb5	56	0.00	0.00	0.03	0.00	0.00	0.00	0.00	0.00	0.00	0.00	0.00	0.00
Immunoglobulin heavy chain variable region, partial	15	0.07	0.02	0.07	0.07	0.07	0.07	0.00	0.00	0.07	0.07	0.00	0.07
Heterogeneous nuclear ribonucleoproteins A2/B1 isoform X4	37	0.01	0.00	0.03	0.03	0.03	0.03	0.00	0.00	0.03	0.00	0.00	0.03
Chain E, Crystal Structure Of Apo Glutamine Synthetase	43	0.01	0.00	0.02	0.02	0.02	0.02	0.02	0.00	0.02	0.00	0.00	0.00
Desmoglein 3	108	0.02	0.01	0.03	0.00	0.00	0.01	0.00	0.00	0.01	0.00	0.00	0.00
60 kDa heat shock protein, mitochondrial isoform X1	61	0.01	0.00	0.02	0.03	0.00	0.03	0.00	0.00	0.00	0.00	0.00	0.00
Plectin isoform X19	518	0.00	0.00	0.00	0.00	0.00	0.00	0.00	0.00	0.00	0.00	0.00	0.00
Isocitrate dehydrogenase [NADP] cytoplasmic isoform 1	47	0.02	0.01	0.02	0.02	0.00	0.02	0.00	0.00	0.00	0.00	0.00	0.00
Phospholipase A2 inhibitor and Ly6/PLAUR domain-containing protein	26	0.00	0.00	0.03	0.00	0.00	0.04	0.00	0.00	0.00	0.00	0.00	0.08
Transglutaminase 1	90	0.01	0.00	0.01	0.00	0.00	0.01	0.00	0.00	0.02	0.00	0.00	0.00
Alpha-2-macroglobulin-like 1	160	0.00	0.00	0.00	0.00	0.00	0.02	0.00	0.00	0.01	0.00	0.00	0.00
Complement factor H isoform 2	140	0.00	0.00	0.00	0.00	0.00	0.00	0.00	0.00	0.00	0.01	0.00	0.02

Protein S100-P	11	0.09	0.09	0.09	0.09	0.09	0.09	0.09	0.00	0.09	0.00	0.00	0.00
EPS8-like 1 isoform X1	83	0.01	0.00	0.01	0.01	0.00	0.01	0.01	0.00	0.01	0.00	0.00	0.00
Epiplakin	321	0.00	0.00	0.01	0.00	0.00	0.00	0.00	0.00	0.00	0.00	0.00	0.00
Ly6/PLAUR domain-containing protein 3 isoform X2	36	0.04	0.01	0.06	0.03	0.00	0.03	0.00	0.00	0.01	0.00	0.00	0.00
Adenosylhomocysteinase isoform 1	48	0.02	0.01	0.02	0.02	0.00	0.06	0.00	0.00	0.01	0.00	0.00	0.00
Leukotriene A-4 hydrolase	69	0.00	0.00	0.01	0.01	0.00	0.01	0.00	0.00	0.01	0.00	0.00	0.01
Calmodulin isoform 2	17	0.00	0.00	0.00	0.00	0.00	0.06	0.00	0.00	0.06	0.00	0.00	0.06
Myosin-14 isoform 2	228	0.00	0.00	0.00	0.00	0.00	0.00	0.00	0.00	0.00	0.00	0.00	0.00
ADP-ribosylation factor 1	21	0.00	0.00	0.04	0.05	0.00	0.10	0.00	0.00	0.05	0.00	0.00	0.00
Gastrokine-1	20	0.00	0.00	0.00	0.00	0.00	0.00	0.00	0.00	0.00	0.05	0.00	0.05
Protein S100-A11	12	0.04	0.00	0.08	0.08	0.08	0.08	0.08	0.04	0.08	0.00	0.00	0.08
Protein S100-A16 isoform X4	12	0.08	0.02	0.08	0.00	0.00	0.08	0.08	0.08	0.08	0.00	0.00	0.00
40S ribosomal protein S3	27	0.00	0.00	0.00	0.00	0.00	0.04	0.00	0.00	0.02	0.00	0.00	0.00
Ceruloplasmin-like	119	0.00	0.00	0.01	0.01	0.01	0.02	0.00	0.00	0.00	0.00	0.00	0.00
Puromycin-sensitive aminopeptidase	97	0.01	0.00	0.01	0.01	0.00	0.01	0.00	0.00	0.01	0.00	0.00	0.00
Fatty acid-binding protein, epidermal	17	0.03	0.00	0.06	0.12	0.00	0.12	0.00	0.00	0.06	0.00	0.00	0.00
Glutathione S-transferase Mu 3 isoform X2	22	0.00	0.00	0.03	0.00	0.00	0.09	0.00	0.00	0.02	0.00	0.00	0.00
Glutathione peroxidase 3 precursor	25	0.00	0.00	0.00	0.00	0.00	0.00	0.00	0.00	0.04	0.00	0.00	0.04
Suprabasin isoform X1	84	0.00	0.00	0.02	0.00	0.00	0.00	0.00	0.00	0.01	0.00	0.00	0.00
Pepsin A preproprotein	42	0.00	0.00	0.00	0.00	0.00	0.00	0.00	0.00	0.00	0.05	0.05	0.07
Chymotrypsin-like	29	0.00	0.00	0.00	0.00	0.00	0.00	0.00	0.00	0.00	0.10	0.00	0.14
Eosinophil peroxidase	75	0.00	0.00	0.00	0.00	0.00	0.04	0.00	0.00	0.00	0.00	0.00	0.00
Histone H2B type 1-J-like	14	0.07	0.00	0.14	0.29	0.21	0.79	0.14	0.00	0.32	0.00	0.00	0.00
Mucin-1 precursor	56	0.02	0.00	0.03	0.00	0.00	0.00	0.00	0.00	0.02	0.02	0.02	0.02
Rho GDP-dissociation inhibitor 1 isoform 2	23	0.04	0.01	0.04	0.04	0.04	0.09	0.00	0.00	0.04	0.00	0.00	0.00
Rab GDP dissociation inhibitor beta	50	0.01	0.00	0.04	0.02	0.00	0.04	0.00	0.00	0.02	0.00	0.00	0.00
Pp90 precursor	68	0.01	0.00	0.01	0.01	0.00	0.03	0.00	0.00	0.00	0.00	0.00	0.00

Peroxiredoxin-5, mitochondrial isoform X1	19	0.00	0.00	0.04	0.05	0.00	0.05	0.00	0.00	0.03	0.00	0.00	0.00
Poly(U)-specific endoribonuclease isoform X4	40	0.00	0.00	0.02	0.03	0.03	0.05	0.00	0.00	0.00	0.00	0.00	0.00
Beta-2-microglobulin	14	0.07	0.02	0.13	0.00	0.00	0.00	0.00	0.00	0.04	0.00	0.00	0.00
Peptidyl-prolyl cis-trans isomerase B isoform 2	24	0.04	0.00	0.08	0.08	0.00	0.08	0.00	0.00	0.00	0.00	0.00	0.00
Trefoil factor family peptide 3	9	0.00	0.00	0.08	0.00	0.00	0.00	0.11	0.00	0.28	0.00	0.00	0.00
Keratin, type II cytoskeletal 75	62	0.00	0.00	0.00	0.00	0.00	0.00	0.16	0.00	0.29	0.00	0.00	0.00
Inter-alpha-trypsin inhibitor heavy chain H4 isoform X1	113	0.00	0.00	0.00	0.00	0.00	0.02	0.00	0.00	0.00	0.00	0.00	0.01
BPI fold-containing family A member 1	27	0.02	0.00	0.04	0.04	0.04	0.04	0.04	0.00	0.04	0.00	0.00	0.04
Cadherin-1	98	0.01	0.01	0.01	0.00	0.00	0.01	0.01	0.00	0.01	0.00	0.00	0.00
Carcinoembryonic antigen-related cell adhesion molecule 1 isoform 4S	49	0.01	0.00	0.02	0.02	0.02	0.02	0.02	0.00	0.02	0.00	0.00	0.00
Synaptic vesicle membrane protein VAT-1 homolog	43	0.02	0.02	0.02	0.02	0.00	0.02	0.00	0.00	0.02	0.00	0.00	0.00
Fetuin-B	42	0.02	0.02	0.02	0.00	0.00	0.02	0.00	0.00	0.00	0.02	0.00	0.02
Thrombospondin-1	130	0.00	0.00	0.01	0.00	0.00	0.00	0.00	0.00	0.01	0.00	0.00	0.01
Elongation factor 1-gamma isoform 1	50	0.00	0.00	0.00	0.02	0.02	0.02	0.00	0.00	0.01	0.00	0.00	0.00
Transmembrane protease serine 11B-like protein-like	47	0.00	0.00	0.02	0.00	0.00	0.02	0.00	0.00	0.02	0.00	0.00	0.00
Retinal dehydrogenase 1 isoform 1	55	0.00	0.00	0.01	0.00	0.00	0.00	0.00	0.00	0.01	0.00	0.00	0.00
Putative V-set and immunoglobulin domain-containing-like protein IGHV4OR15-8-like	22	0.00	0.00	0.00	0.00	0.00	0.05	0.00	0.00	0.00	0.00	0.00	0.05
Peroxiredoxin-2 isoform 1	22	0.00	0.00	0.00	0.00	0.00	0.00	0.00	0.00	0.05	0.00	0.00	0.00
Olfactomedin-4	49	0.00	0.00	0.00	0.00	0.00	0.02	0.00	0.00	0.00	0.00	0.00	0.00
Voltage-dependent anion-selective channel protein 2 isoform 2	32	0.00	0.00	0.02	0.03	0.03	0.03	0.00	0.00	0.03	0.00	0.00	0.00
Chain A, Structure Of Full Length Grp94 With Amp-Pnp Bound	93	0.00	0.00	0.01	0.00	0.00	0.00	0.00	0.00	0.00	0.00	0.00	0.01
Ferritin light chain-like	20	0.03	0.00	0.05	0.05	0.00	0.05	0.00	0.00	0.05	0.00	0.00	0.00

Tubulin alpha-4A chain isoform X2	48	0.00	0.00	0.03	0.10	0.10	0.10	0.00	0.00	0.02	0.00	0.00	0.00
WD repeat domain 1	66	0.01	0.00	0.02	0.00	0.00	0.03	0.00	0.00	0.00	0.00	0.00	0.00
Protease inhibitor	14	0.00	0.00	0.11	0.00	0.00	0.00	0.00	0.00	0.04	0.00	0.00	0.14
Deoxyribonuclease I	31	0.00	0.00	0.00	0.00	0.00	0.03	0.00	0.00	0.00	0.03	0.00	0.06
Angiotensinogen	52	0.00	0.00	0.00	0.00	0.00	0.02	0.00	0.00	0.00	0.00	0.00	0.00
Submaxillary gland androgen regulated protein 3A isoformX1	30	0.00	0.00	0.00	0.00	0.00	0.03	0.00	0.00	0.00	0.00	0.00	0.00
Afamin	69	0.00	0.00	0.00	0.00	0.00	0.00	0.00	0.00	0.00	0.00	0.00	0.03
Chymosin-like	42	0.00	0.00	0.00	0.00	0.00	0.00	0.00	0.00	0.00	0.05	0.05	0.07
Guanine nucleotide-binding protein G(t) subunit alpha-1	41	0.00	0.00	0.00	0.00	0.00	0.05	0.00	0.00	0.01	0.00	0.00	0.00
Integrin beta-2 isoform X1	85	0.00	0.00	0.00	0.00	0.00	0.02	0.00	0.00	0.00	0.00	0.00	0.00
Arachidonate 15-lipoxygenase	76	0.00	0.00	0.00	0.00	0.00	0.04	0.00	0.00	0.00	0.00	0.00	0.00
Complement C5	180	0.00	0.00	0.00	0.00	0.00	0.00	0.00	0.00	0.00	0.00	0.00	0.00
Type 2A protein phosphatase catalytic subunit	36	0.01	0.00	0.03	0.03	0.00	0.03	0.00	0.00	0.01	0.00	0.00	0.00
Serine/arginine repetitive matrix protein 3	49	0.01	0.00	0.02	0.02	0.00	0.04	0.00	0.00	0.01	0.00	0.00	0.00
Annexin A4	36	0.03	0.01	0.03	0.00	0.00	0.06	0.00	0.00	0.01	0.00	0.00	0.00
Peptidoglycan recognition protein 1	22	0.00	0.00	0.03	0.05	0.00	0.05	0.00	0.00	0.02	0.00	0.00	0.00
Cystatin-M	16	0.00	0.00	0.05	0.00	0.00	0.06	0.00	0.00	0.06	0.00	0.00	0.00
Tubulin beta chain	50	0.06	0.00	0.15	0.10	0.00	0.14	0.00	0.00	0.09	0.00	0.00	0.00
Immunoglobulin heavy chain constant region CH1	11	0.05	0.00	0.09	0.00	0.00	0.00	0.00	0.00	0.00	0.00	0.00	0.09
ADP-ribosylation factor 6	20	0.00	0.00	0.04	0.00	0.00	0.00	0.00	0.00	0.05	0.00	0.00	0.00
Glutathione S-transferase pi 1	24	0.00	0.00	0.00	0.00	0.00	0.04	0.00	0.00	0.04	0.00	0.00	0.00
Immunoglobulin alpha heavy chain constant regin variant D	9	0.00	0.00	0.50	0.00	0.00	0.33	0.00	0.00	0.00	0.00	0.00	0.00
TPA: predicted NADPH oxidase-2	65	0.00	0.00	0.00	0.00	0.00	0.05	0.00	0.00	0.01	0.00	0.00	0.00
Myosin-7	223	0.00	0.00	0.00	0.00	0.00	0.00	0.00	0.00	0.00	0.00	0.00	0.00
Carbamoyl-phosphate synthase [ammonia], mitochondrial isoform 1	165	0.00	0.00	0.00	0.00	0.00	0.00	0.00	0.00	0.00	0.00	0.00	0.00

Phosphatidylinositol-glycan-specific phospholipase D isoform X2	93	0.00	0.00	0.00	0.00	0.00	0.00	0.00	0.00	0.00	0.00	0.00	0.01
Ubiquitin-like protein ISG15	19	0.05	0.01	0.05	0.00	0.00	0.05	0.05	0.00	0.05	0.00	0.00	0.00
Kallikrein-related peptidase 10	30	0.00	0.00	0.03	0.00	0.00	0.03	0.00	0.00	0.03	0.00	0.00	0.00
Translationally-controlled tumor protein	20	0.00	0.00	0.04	0.00	0.00	0.05	0.00	0.00	0.05	0.00	0.00	0.00
Nucleoside diphosphate kinase A-like	17	0.00	0.00	0.04	0.06	0.00	0.12	0.00	0.00	0.03	0.00	0.00	0.00
Ubiquitin-like modifier activating enzyme 1	120	0.00	0.00	0.01	0.00	0.00	0.00	0.01	0.00	0.01	0.00	0.00	0.00
Coactosin-like protein, partial	14	0.00	0.00	0.05	0.00	0.00	0.14	0.00	0.00	0.04	0.00	0.00	0.00
Uridine phosphorylase 1	38	0.00	0.00	0.02	0.03	0.00	0.05	0.00	0.00	0.00	0.00	0.00	0.00
Hexokinase-3	104	0.00	0.00	0.01	0.00	0.00	0.01	0.00	0.00	0.00	0.00	0.00	0.00
C4b-binding protein alpha chain isoform X1	69	0.00	0.00	0.00	0.00	0.00	0.01	0.00	0.00	0.00	0.00	0.00	0.00
Succinyl-CoA:3-ketoacid coenzyme A transferase 1, mitochondrial	65	0.02	0.00	0.02	0.00	0.00	0.00	0.00	0.00	0.00	0.00	0.00	0.00
Copine-3	60	0.00	0.00	0.00	0.00	0.00	0.00	0.00	0.00	0.00	0.00	0.00	0.00
DNA dC->dU-editing enzyme APOBEC-3A	23	0.00	0.00	0.00	0.00	0.00	0.00	0.00	0.00	0.04	0.00	0.00	0.00
Mucin-6-like, partial	140	0.00	0.00	0.00	0.00	0.00	0.00	0.00	0.00	0.00	0.01	0.01	0.01
Keratin, type II cuticular Hb1 isoform 1	56	0.00	0.00	0.00	0.00	0.00	0.00	0.00	0.00	0.01	0.00	0.00	0.00
Carbonic anhydrase 1	29	0.00	0.00	0.00	0.00	0.00	0.00	0.00	0.00	0.00	0.00	0.00	0.00
Keratin, type II cytoskeletal 1b	61	0.00	0.00	0.00	0.00	0.00	0.00	0.00	0.00	0.00	0.00	0.00	0.00
Histidine-rich glycoprotein isoform X1	62	0.00	0.00	0.01	0.00	0.00	0.00	0.00	0.00	0.02	0.00	0.00	0.02
Anterior gradient protein 2 homolog isoformX1	20	0.00	0.00	0.00	0.00	0.00	0.00	0.05	0.00	0.05	0.05	0.00	0.10
Protein-arginine deiminase type-1 isoform 1	74	0.01	0.00	0.01	0.00	0.00	0.00	0.00	0.00	0.01	0.00	0.00	0.00
Complement factor I isoform X4	67	0.00	0.00	0.00	0.00	0.00	0.01	0.00	0.00	0.00	0.00	0.00	0.03
Transforming protein RhoA	22	0.00	0.00	0.00	0.00	0.00	0.09	0.00	0.00	0.02	0.00	0.00	0.00
Serine/arginine-rich splicing factor 3	19	0.00	0.00	0.00	0.05	0.00	0.05	0.00	0.00	0.03	0.00	0.00	0.00

Dipeptidyl peptidase 1	49	0.00	0.00	0.02	0.00	0.00	0.02	0.00	0.00	0.00	0.00	0.00	0.00
Tropomyosin alpha-3 chain isoform X7	29	0.00	0.00	0.00	0.00	0.00	0.03	0.00	0.00	0.02	0.00	0.00	0.00
Heterogeneous nuclear ribonucleoprotein D-like	38	0.00	0.00	0.00	0.00	0.00	0.00	0.00	0.00	0.03	0.00	0.00	0.00
Phosphatidylethanolamine-binding protein 4 isoformX1	26	0.00	0.00	0.00	0.00	0.00	0.00	0.00	0.00	0.02	0.00	0.00	0.04
Keratin, type II cytoskeletal 80	51	0.00	0.00	0.06	0.00	0.00	0.18	0.00	0.00	0.00	0.00	0.00	0.00
Ras-related C3 botulinum toxin substrate 2	21	0.02	0.00	0.05	0.00	0.00	0.00	0.00	0.00	0.00	0.00	0.00	0.00
Caspase-14	28	0.00	0.00	0.00	0.00	0.00	0.00	0.00	0.00	0.00	0.00	0.00	0.00
Transcobalamin-1	48	0.00	0.00	0.00	0.00	0.00	0.00	0.00	0.00	0.00	0.02	0.00	0.06
Resistin like beta	12	0.00	0.00	0.00	0.00	0.00	0.00	0.00	0.00	0.00	0.00	0.00	0.08
Annexin A10	52	0.00	0.00	0.00	0.00	0.00	0.00	0.00	0.00	0.00	0.02	0.00	0.04
Vitelline membrane outer layer protein 1 homolog isoform X2	23	0.00	0.00	0.00	0.00	0.00	0.00	0.00	0.00	0.00	0.00	0.00	0.00
Alpha-1-antichymotrypsin isoform X1	47	0.00	0.00	0.00	0.00	0.00	0.00	0.00	0.00	0.00	0.00	0.00	0.00
Xaa-Pro dipeptidase isoformX1	48	0.00	0.00	0.02	0.02	0.02	0.02	0.00	0.00	0.00	0.00	0.00	0.00
Immunoglobulin lambda light chain variable region	13	0.00	0.00	0.06	0.00	0.00	0.00	0.00	0.00	0.04	0.08	0.00	0.08
Allergen Can f 4 isoform X1	19	0.03	0.00	0.13	0.00	0.00	0.00	0.00	0.00	0.03	0.00	0.00	0.00
Mammaglobin-A	11	0.00	0.00	0.07	0.00	0.00	0.00	0.00	0.00	0.00	0.00	0.00	0.00
D-beta-hydroxybutyrate dehydrogenase, mitochondrial	38	0.00	0.00	0.00	0.00	0.00	0.03	0.00	0.00	0.00	0.00	0.00	0.00
Proteasome subunit alpha type-6 isoformX2	27	0.00	0.00	0.00	0.00	0.00	0.04	0.00	0.00	0.04	0.00	0.00	0.00
Clathrin heavy chain 1 isoform X2	192	0.00	0.00	0.00	0.00	0.00	0.00	0.01	0.00	0.01	0.00	0.00	0.00
Inhibitor of carbonic anhydrase-like	77	0.00	0.00	0.00	0.00	0.00	0.00	0.00	0.00	0.00	0.00	0.00	0.01
Alpha-2-antiplasmin isoform X3	62	0.00	0.00	0.00	0.00	0.00	0.00	0.00	0.00	0.00	0.00	0.00	0.02
Keratin, type II cytoskeletal 2 oral	67	0.00	0.00	0.00	0.00	0.00	0.45	0.00	0.00	0.00	0.00	0.00	0.00
Carbonic anhydrase 2	29	0.00	0.00	0.00	0.00	0.00	0.00	0.00	0.00	0.00	0.00	0.00	0.03
Keratin, type I cuticular Ha8	51	0.00	0.00	0.00	0.00	0.00	0.00	0.00	0.00	0.00	0.00	0.00	0.00
60S acidic ribosomal protein P2	12	0.00	0.00	0.06	0.00	0.00	0.08	0.00	0.00	0.00	0.00	0.00	0.00

Fatty acid synthase	269	0.00	0.00	0.00	0.00	0.00	0.00	0.00	0.00	0.00	0.00	0.00	0.00
Ras-related protein Rab-11B	24	0.00	0.00	0.00	0.00	0.00	0.04	0.00	0.00	0.00	0.00	0.00	0.00
Phosphoglucomutase-1 isoform 1	64	0.00	0.00	0.01	0.00	0.00	0.00	0.00	0.00	0.00	0.00	0.00	0.00
Stress-70 protein, mitochondrial isoform 6	74	0.00	0.00	0.00	0.00	0.00	0.00	0.00	0.00	0.01	0.00	0.00	0.00
Keratin, type II cytoskeletal 2 epidermal isoform X2	61	0.00	0.00	0.00	0.44	0.00	0.57	0.00	0.00	0.00	0.00	0.00	0.00
Keratin, type I cytoskeletal 23 isoform X1	48	0.00	0.00	0.00	0.00	0.00	0.02	0.00	0.00	0.00	0.00	0.00	0.00
Protein-arginine deiminase type-2	81	0.00	0.00	0.00	0.00	0.00	0.02	0.00	0.00	0.00	0.00	0.00	0.00
Band 3 anion transport protein	104	0.00	0.00	0.00	0.00	0.00	0.00	0.00	0.00	0.00	0.00	0.00	0.01
Vinculin	127	0.00	0.00	0.00	0.00	0.00	0.01	0.00	0.00	0.00	0.00	0.00	0.00
Gastrokine-2	21	0.00	0.00	0.00	0.00	0.00	0.00	0.00	0.00	0.00	0.00	0.00	0.14
Spectrin alpha chain, erythrocytic 1	273	0.00	0.00	0.00	0.00	0.00	0.00	0.00	0.00	0.00	0.00	0.00	0.00
Keratin-associated protein 13-2	18	0.00	0.00	0.00	0.00	0.00	0.11	0.00	0.00	0.08	0.00	0.00	0.00
Tetraspanin-8	26	0.00	0.00	0.00	0.00	0.00	0.00	0.00	0.00	0.00	0.04	0.04	0.04
Talin-1 isoform X1	270	0.00	0.00	0.00	0.00	0.00	0.00	0.00	0.00	0.00	0.00	0.00	0.00
Transmembrane emp24 domain-containing protein 9 isoform 1	27	0.00	0.00	0.03	0.00	0.00	0.04	0.00	0.00	0.00	0.00	0.00	0.00
DDX3Y	73	0.00	0.00	0.00	0.00	0.00	0.01	0.00	0.00	0.01	0.00	0.00	0.00
Myosin light chain 1/3, skeletal muscle isoform isoformX1	17	0.00	0.00	0.00	0.00	0.00	0.06	0.00	0.00	0.00	0.00	0.00	0.00
Citrate synthase, mitochondrial isoform 2	52	0.00	0.00	0.00	0.00	0.00	0.02	0.00	0.00	0.00	0.00	0.00	0.00
Tripartite motif-containing protein 29	65	0.00	0.00	0.00	0.00	0.00	0.00	0.00	0.00	0.00	0.00	0.00	0.00
Plasma PAF acetylhydrolase	50	0.00	0.00	0.00	0.02	0.00	0.04	0.00	0.00	0.00	0.00	0.00	0.00
Glutamate dehydrogenase 1, mitochondrial	65	0.00	0.00	0.00	0.00	0.00	0.00	0.00	0.00	0.00	0.00	0.00	0.00
Spectrin alpha chain, non-erythrocytic 1 isoform X1	289	0.00	0.00	0.00	0.00	0.00	0.00	0.00	0.00	0.00	0.00	0.00	0.00
Receptor-type tyrosine-protein phosphatase C isoform X1	148	0.00	0.00	0.00	0.00	0.00	0.00	0.00	0.00	0.00	0.00	0.00	0.00
Hemoglobin subunit epsilon-2	16	0.00	0.00	0.00	0.00	0.00	0.06	0.00	0.00	0.00	0.00	0.00	0.00
Heterogeneous nuclear ribonucleoprotein U	80	0.00	0.00	0.00	0.00	0.00	0.01	0.00	0.00	0.00	0.00	0.00	0.00

Alpha-actinin-1 isoformX2	103	0.00	0.00	0.00	0.00	0.00	0.06	0.00	0.00	0.00	0.00	0.00	0.00
Secreted phosphoprotein 24 isoform X1	23	0.00	0.00	0.00	0.00	0.00	0.00	0.00	0.00	0.02	0.00	0.00	0.00
Flavin reductase (NADPH) isoform X2	22	0.00	0.00	0.00	0.00	0.00	0.00	0.00	0.00	0.00	0.00	0.00	0.00
Keratin-associated protein 14-like	18	0.00	0.00	0.00	0.00	0.00	0.00	0.00	0.00	0.00	0.00	0.00	0.00
Trichohyalin	187	0.00	0.00	0.00	0.00	0.00	0.00	0.00	0.00	0.00	0.00	0.00	0.00
Selenium-binding protein 1	52	0.00	0.00	0.00	0.00	0.00	0.00	0.00	0.00	0.00	0.00	0.00	0.02
Fast myosin heavy chain 2B	223	0.00	0.00	0.00	0.00	0.00	0.00	0.00	0.00	0.00	0.00	0.00	0.00
CD5 molecule-like	38	0.00	0.00	0.00	0.00	0.00	0.00	0.00	0.00	0.00	0.00	0.00	0.00
Developmental myosin heavy chain neonatal	223	0.00	0.00	0.00	0.00	0.00	0.00	0.00	0.00	0.00	0.00	0.00	0.00
V-type proton ATPase subunit B, brain isoform isoform 1	57	0.00	0.00	0.00	0.00	0.00	0.02	0.00	0.00	0.01	0.00	0.00	0.00
Neutral alpha-glucosidase AB isoformX2	106	0.00	0.00	0.00	0.01	0.00	0.01	0.00	0.00	0.00	0.00	0.00	0.00
Alpha-crystallin B chain isoform 5	20	0.00	0.00	0.00	0.00	0.00	0.00	0.00	0.00	0.03	0.00	0.00	0.00
Folate receptor beta isoform X2	30	0.00	0.00	0.00	0.00	0.00	0.03	0.00	0.00	0.00	0.00	0.00	0.00
Protein disulfide-isomerase A4 isoform 3	72	0.00	0.00	0.00	0.00	0.00	0.00	0.00	0.00	0.00	0.00	0.00	0.00
ERO1-like protein alpha	54	0.00	0.00	0.00	0.00	0.00	0.02	0.00	0.00	0.00	0.00	0.00	0.00
Trefoil factor family peptide 1	9	0.00	0.00	0.00	0.00	0.00	0.00	0.00	0.00	0.00	0.11	0.00	0.11
Alpha-actinin-2 isoform X4	105	0.00	0.00	0.00	0.00	0.00	0.00	0.00	0.00	0.00	0.00	0.00	0.00
Keratin, type II microfibrillar, component 7C	54	0.00	0.00	0.00	0.00	0.00	0.00	0.00	0.00	0.00	0.00	0.00	0.00
Filaggrin isoform X1	313	0.00	0.00	0.00	0.00	0.00	0.00	0.00	0.00	0.00	0.00	0.00	0.00
Keratin, type I cuticular Ha1	46	0.00	0.00	0.00	0.00	0.00	0.00	0.00	0.00	0.00	0.00	0.00	0.00
Serine/threonine-protein phosphatase 2A 65 kDa regulatory subunit A alpha isoform isoform X3	65	0.00	0.00	0.00	0.00	0.00	0.00	0.00	0.00	0.01	0.00	0.00	0.00
Maltase-glucoamylase, intestinal	207	0.00	0.00	0.00	0.00	0.00	0.00	0.00	0.00	0.00	0.00	0.00	0.00
6-phosphogluconolactonase	28	0.00	0.00	0.00	0.00	0.00	0.04	0.00	0.00	0.02	0.00	0.00	0.00
Glutathione S-transferase A2 isoform 2	26	0.00	0.00	0.00	0.00	0.00	0.00	0.00	0.00	0.00	0.00	0.00	0.04

BTB/POZ domain-containing protein KCTD12	36	0.00	0.00	0.00	0.00	0.00	0.03	0.00	0.00	0.00	0.00	0.00	0.00
Protein FAM49B isoformX1	37	0.00	0.00	0.00	0.00	0.00	0.03	0.00	0.00	0.00	0.00	0.00	0.00
Proactivator polypeptide isoform X7	59	0.00	0.00	0.00	0.00	0.00	0.00	0.00	0.00	0.00	0.00	0.00	0.02
Syncollin	15	0.00	0.00	0.00	0.00	0.00	0.00	0.00	0.00	0.00	0.00	0.00	0.07
Keratin-associated protein 7-1	10	0.00	0.00	0.00	0.00	0.00	0.00	0.00	0.00	0.00	0.00	0.00	0.00
Insulin-like growth factor binding protein 2, 36kDa, partial	27	0.00	0.00	0.00	0.00	0.00	0.00	0.00	0.00	0.00	0.00	0.00	0.04
Secretoglobin family 1C member 1	10	0.00	0.00	0.00	0.00	0.00	0.00	0.00	0.00	0.00	0.00	0.00	0.00
Hexokinase-1 isoform X5	103	0.00	0.00	0.00	0.00	0.00	0.01	0.00	0.00	0.00	0.00	0.00	0.00
Epidermal growth factor	134	0.00	0.00	0.01	0.00	0.00	0.00	0.00	0.00	0.00	0.00	0.00	0.00
CD59 glycoprotein isoform X1	14	0.00	0.00	0.00	0.00	0.00	0.00	0.00	0.00	0.00	0.00	0.00	0.07
Heterogeneous nuclear ribonucleoprotein H2 isoformX7	49	0.00	0.00	0.00	0.00	0.00	0.00	0.00	0.00	0.02	0.00	0.00	0.00
ADP-ribosylation factor 4 isoform 2	21	0.00	0.00	0.00	0.00	0.00	0.00	0.00	0.00	0.00	0.00	0.00	0.00
BAT1	49	0.00	0.00	0.00	0.00	0.00	0.00	0.00	0.00	0.01	0.00	0.00	0.00
Adipocyte plasma membrane-associated protein	46	0.00	0.00	0.00	0.00	0.00	0.00	0.00	0.00	0.00	0.00	0.00	0.00
Pancreatic intrinsic factor	45	0.00	0.00	0.00	0.00	0.00	0.00	0.00	0.00	0.00	0.00	0.00	0.02
Keratin, type I cuticular Ha3-I	46	0.00	0.00	0.00	0.00	0.00	0.00	0.00	0.00	0.00	0.00	0.00	0.00
Cytochrome c oxidase subunit 5A, mitochondrial	17	0.00	0.00	0.00	0.00	0.00	0.00	0.00	0.00	0.03	0.00	0.00	0.00
Fermitin family homolog 3	75	0.00	0.00	0.00	0.00	0.00	0.00	0.00	0.00	0.00	0.00	0.00	0.00
keratin-associated protein 13-2	18	0.00	0.00	0.00	0.00	0.00	0.00	0.00	0.00	0.00	0.00	0.00	0.00
Adenylate kinase 1	23	0.00	0.00	0.00	0.00	0.00	0.00	0.00	0.00	0.00	0.00	0.00	0.00
Keratin, type I cuticular Ha8	49	0.00	0.00	0.00	0.00	0.00	0.00	0.00	0.00	0.00	0.00	0.00	0.00
CD63 antigen isoformX1	26	0.00	0.00	0.00	0.00	0.00	0.00	0.00	0.00	0.00	0.00	0.00	0.00
Antimicrobial peptide NK-lysin-like	18	0.00	0.00	0.00	0.00	0.00	0.00	0.00	0.00	0.00	0.00	0.00	0.00
Unnamed protein product	52	0.00	0.00	0.00	0.00	0.00	0.00	0.00	0.00	0.00	0.00	0.00	0.00
Lamin-B1 isoform X6	52	0.00	0.00	0.00	0.00	0.00	0.00	0.00	0.00	0.00	0.00	0.00	0.00
Fructose-1,6-bisphosphatase 1 isoform X4	31	0.00	0.00	0.00	0.00	0.00	0.00	0.00	0.00	0.00	0.00	0.00	0.00

Acidic leucine-rich nuclear phosphoprotein 32 family member B isoform X2	29	0.00	0.00	0.00	0.00	0.00	0.03	0.00	0.00	0.00	0.00	0.00	0.00
Ras-related protein Rap-1b isoform 2	21	0.00	0.00	0.00	0.00	0.00	0.00	0.00	0.00	0.00	0.00	0.00	0.00
Protein FAM3C	25	0.00	0.00	0.00	0.00	0.00	0.00	0.00	0.00	0.00	0.00	0.00	0.00
Plasminogen activator inhibitor 2	46	0.00	0.00	0.00	0.00	0.00	0.00	0.00	0.00	0.00	0.00	0.00	0.00
TPA: predicted neutrophil cytosolic factor 1	38	0.00	0.00	0.00	0.00	0.00	0.00	0.00	0.00	0.00	0.00	0.00	0.00
Spectrin beta chain, erythrocyte	247	0.00	0.00	0.00	0.00	0.00	0.00	0.00	0.00	0.00	0.00	0.00	0.00
Angiotensin-converting enzyme	151	0.00	0.00	0.00	0.00	0.00	0.00	0.00	0.00	0.00	0.00	0.00	0.00
Apolipoprotein A-IV	44	0.00	0.00	0.00	0.00	0.00	0.00	0.00	0.00	0.00	0.00	0.00	0.00
Serpin B4 isoform 1	45	0.00	0.00	0.00	0.00	0.00	0.00	0.00	0.00	0.00	0.00	0.00	0.00
Tropomyosin alpha-4 chain isoform X8	26	0.00	0.00	0.00	0.00	0.00	0.00	0.00	0.00	0.00	0.00	0.00	0.00
Galectin-1	15	0.00	0.00	0.00	0.00	0.00	0.00	0.00	0.00	0.00	0.00	0.00	0.00
Protein AMBP	39	0.00	0.00	0.00	0.00	0.00	0.00	0.00	0.00	0.00	0.00	0.00	0.00
Myosin light chain 3	22	0.00	0.00	0.00	0.00	0.00	0.00	0.00	0.00	0.00	0.00	0.00	0.00
Mucin-13	61	0.00	0.00	0.00	0.00	0.00	0.00	0.00	0.00	0.00	0.00	0.00	0.00
Tartrate-resistant acid phosphatase type 5 isoform X2	38	0.00	0.00	0.00	0.00	0.00	0.00	0.00	0.00	0.00	0.00	0.00	0.00
NADH cytochrome b5 reductase	34	0.00	0.00	0.00	0.00	0.00	0.00	0.00	0.00	0.00	0.00	0.00	0.00

Supplemental Table 1: All detected proteins from each site are provided. Protein concentrations are displayed as median (IQR) normalized spectral abundance factors (NSAF). Molecular weight for each protein is also provided (kDA).
